# Sara phosphorylation state controls the dispatch of endosomes from the central spindle during asymmetric division

**DOI:** 10.1038/ncomms15285

**Published:** 2017-06-06

**Authors:** Sylvain Loubéry, Alicia Daeden, Carole Seum, Laurent Holtzer, Ana Moraleda, Nicolas Damond, Emmanuel Derivery, Thomas Schmidt, Marcos Gonzalez-Gaitan

**Affiliations:** 1 Department of Biochemistry, University of Geneva, Geneva 1211, Switzerland

## Abstract

During asymmetric division, fate assignation in daughter cells is mediated by the partition of determinants from the mother. In the fly sensory organ precursor cell, Notch signalling partitions into the pIIa daughter. Notch and its ligand Delta are endocytosed into Sara endosomes in the mother cell and they are first targeted to the central spindle, where they get distributed asymmetrically to finally be dispatched to pIIa. While the processes of endosomal targeting and asymmetry are starting to be understood, the machineries implicated in the final dispatch to pIIa are unknown. We show that Sara binds the PP1c phosphatase and its regulator Sds22. Sara phosphorylation on three specific sites functions as a switch for the dispatch: if not phosphorylated, endosomes are targeted to the spindle and upon phosphorylation of Sara, endosomes detach from the spindle during pIIa targeting.

## Abstract (web summary)

Asymmetric segregation of cell fate determinants during cell division governs daughter cell fate. Here the authors show that Sara endosomes, known to regulate Notch signalling, are targeted to the mitotic spindle and once phosphorylated are asymmetrically dispatched into a daughter cell to determine cell fate.

Asymmetric cell division plays many roles in development. In particular, stem cells divide asymmetrically to self-renew while also forming differentiated cells. Asymmetric cell division involves the specific partitioning of cell fate determinants (RNA, proteins or organelles) in one of the two sibling daughter cells. The Sensory Organ Precursor cells (SOPs) of the *Drosophila* notum are a model system of choice to unravel the molecular mechanisms of asymmetric cell division (reviewed in ref. 1).

The division of each SOP gives rise to a pIIa and a pIIb daughter cell and, after two more rounds of asymmetric cell divisions, to the four cells of the sensory organ: the outer cells (shaft and socket) are progeny of the pIIa, while the pIIb forms the inner cells (sheath and neuron) and a glial cell that rapidly undergoes apoptosis^2^,^3^. The Notch signalling pathway controls cell fate determination in this system: a signalling bias between the pIIa–pIIb sibling cells is essential to obtain a correct lineage.

The asymmetric dispatch of cell fate determinants during SOP division is governed by the polarity of the dividing cell. The Par complex (composed by the aPKC, Par-3 and Par-6 proteins) is the master regulator of the establishment of this polarity (reviewed in ref. 4). Downstream the Par complex, Notch signalling is regulated by endocytosis and endosomal trafficking through four independent mechanisms (reviewed in ref. 5): (1) The E3 Ubiquitin ligase Neuralized is segregated to the pIIb cell, where it induces the endocytosis and thereby the activation of the Notch ligand Delta^6^; (2) Recycling endosomes accumulate in the perinuclear region of the pIIb cell, in which they enhance the recycling and activation of Delta^7^; (3) The endocytic proteins α-adaptin and Numb are segregated to the pIIb cell, where they inhibit the Notch activator Sanpodo^8^,^9^; (4) During SOP mitosis, Sara endosomes transport a signalling pool of Notch and Delta to the pIIa cell, where Notch can be activated^10^,^11^. Asymmetric Sara endosomes have also been shown to operate in the larval neural stem cells^10^ as well as in the adult intestinal stem cells in flies^12^, where they also play a role during asymmetric Notch signalling. In fish, Sara endosomes mediate asymmetric cell fate assignation mediated by Notch during the mitosis of neural precursor of the spinal cord^13^.

Sara endosomes are a subpopulation of Rab5-positive early endosomes characterised by the presence of the endocytic protein Sara. Sara directly binds the lipid phosphatidyl-inositol-3-phosphate and both molecules are found at the surface of these endosomes^14^. We have previously established a pulse-chase antibody uptake assay to monitor the trafficking of endogenous internalised Notch and Delta and showed that both Notch and Delta traffic through Sara endosomes^10^,^11^,^15^. Furthermore, it was shown that Sara endosomes are specifically targeted to the pIIa cell during SOP division, mediating thus the transport of a pool of Notch and Delta that contribute to the activation of Notch in the pIIa. The Notch cargo and its Uninflatable binding partner are required for this asymmetric dispatch^11^. Targeting of Sara endosomes to the central spindle is mediated by a plus-end-directed kinesin, Klp98A (ref. 15). The asymmetric distribution of endosomes at the central spindle results from a higher density of microtubules in pIIb with their plus ends pointed towards pIIa^15^.

Here we show that the Sara protein itself controls both the targeting and the final dispatch of Sara endosomes to the pIIa daughter cell. We find that Sara binds and is a target of the PP1 phosphatase complex. The phosphorylation state of Sara functions as a switch that enables the targeting of Sara endosomes to the central spindle of the dividing SOP, and their subsequent detachment from the central spindle, which is necessary to allow their movement to the pIIa daughter cell.

## Results and Discussion

We have previously shown that a subpopulation of Rab5 early endosomes positive for Sara are asymmetrically dispatched into the pIIa daughter cell during cytokinesis of the SOP^10^,^11^. This was monitored by following *in vivo* either GFP-Sara or internalized Delta or Notch, which reach the Sara endosomes 20 min after their endocytosis in the mother cell^11^,^15^. We termed these vesicles iDelta^20′^ endosomes. In contrast, the pools of Notch in endosomal populations upstream or downstream of the Sara endosomes (that is, the Rab5 early endosomes with low Sara levels and the Rab7 late endosomes, respectively) were segregated symmetrically^10^,^11^. Rab5 endosomes show different levels of Sara signal: by a progressive targeting of Sara to the Rab5 endosomes, Rab5 early endosomes mature into Sara endosomes. This prompts the question whether the levels of Sara in endosomes correlate indeed with their asymmetric behaviour.

### Sara levels correlate with endosomal behaviour

**Figure 1:**
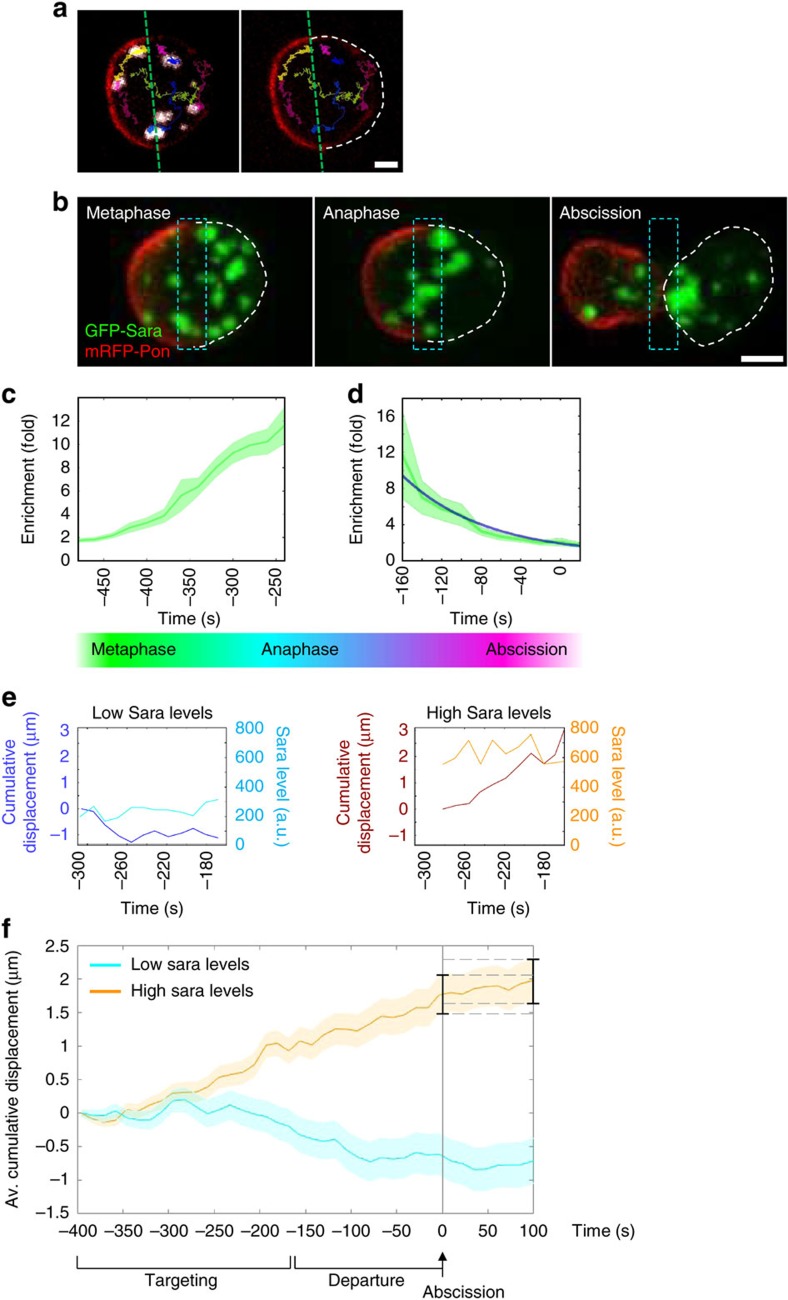
Endosomal central spindle targeting and asymmetric segregation correlates with Sara levels at endosomes. (**a**) Representative tracks of Sara endosome centroids in a metaphase SOP. Left, GFP-Sara (grey levels) and mRFP-Pon (red); the green dashed line forecasts pIIa/pIIb interface as determined by cortical mRFP-Pon localization. Scale bar, 2.5 μm. Right, same tracks without the endosomal signal. The dashed white line outlines the region of the anterior cortical pole only and forecasts the pIIa side. See also the corresponding Supplementary Movie 1. (**b**) Images from a time-lapse movie (*z*-projections) of a dividing SOP in metaphase, anaphase and at abscission. Dashed cyan box, 2 μm-wide region where the central spindle enrichment is measured in **c**. Scale bar, 3 μm. (**c**) Enrichment of GFP-Sara endosomes measured in the 2 μm-wide central spindle area (*n*=12–26 cells in *N*=5 animals). The curve represents average values (sampled every 20 s) and the shaded area represents the s.e.m. In abscises, time is with respect to abscission (*t*=0 s). (**d**) Enrichment of GFP-Sara endosomes measured in the 2 μm-wide central spindle area (*n*=31, *N*=3; in green). The blue line represents the exponential fit used to estimate the residence time. (**e**) Cumulative displacement (dark colours) and Sara levels (light colours) of two representative endosomes with low and high Sara levels, respectively, as in **f**. (**f**) Cumulative average displacement (±s.e.m.) towards the central spindle region per Sara endosome for endosomes of low and high GFP-Sara levels (*n*=20–45 endosomes in one cell). These two different collections of endosomes (high versus low Sara level) were established from the full data set of tracks by segmenting them according to the levels of Sara into two equally sized groups (see Supplementary Methods ‘GFP Sara levels' for details). Note that the data sets in **c**,**d** are different from **a**,**e**,**f**. The timing of the targeting of the endosomes to the spindle (−400 to −160 s) and the departure phase (−160 to 0) are indicated. Abscission corresponds to time 0 s. Note that directed targeting to the spindle of endosomes containing high Sara levels stops after abscission: the cumulative displacement at abscission and 100 s after abscission are not significantly different (error bars overlap as illustrated by the dashed lines). All error bars represent s.e.m.

To study the relationship between the levels of Sara in endosomes and their targeting to the spindle, we first wrote Matlab codes to perform automatic 3D-tracking of the Sara endosomes. Sara endosomes were detected by monitoring a GFP-Sara fusion, which was overexpressed through the UAS/Gal4 system. This way, we monitored the position of the endosomes, their displacement towards and away from the central spindle as well as the levels of Sara. In addition, we detected automatically the position of the Pon cortical crescent, which forecasts the side of the cell that will become the pIIb cell (Fig. 1a; Supplementary Fig. 1; Supplementary Movie 1; for details of the tracking algorithms, see Supplementary Methods).

We studied the localization of endosomes with respect to a 2 μm-wide box centred in the central spindle during SOP mitosis. We measured the enrichment of endosomes in this central spindle as a function of time (Supplementary Methods). We observed two phases in the movement of the endosomes during mitosis: (i) targeting to the central spindle (Fig. 1b,c) and (ii) departure into the pIIa cell (Fig. 1b,d). Figure 1b,c shows that the endosomes are progressively accumulating in the central spindle area from the end of metaphase (∼450 s before abscission) through anaphase and during cytokinesis until they are enriched at the central spindle by about 10-fold at 250 s before abscission.

Subsequently, the endosomes depart from the central spindle area into the pIIa cell (Fig. 1b–d). By fitting an exponential decay to the profile of abundance of the endosomes at the central spindle, we measured the characteristic residence time of the endosomes at the central spindle after the recruitment phase (Supplementary Methods): after recruitment, endosomes remain at the central spindle 98±9.8 s before they depart into one of the daughter cells, preferentially the pIIa cell (Fig. 1d; *n*=17 cells).

We then studied the correlation between central spindle targeting and the abundance of Sara at the endosomes. Automatic 3D-tracking of the Sara endosomes shows that, during late anaphase, endosomes containing high levels of Sara are displaced towards the central spindle (see Fig. 1e for an example and Fig. 1f for the average behaviour of endosomes in a cell (*n*=20–45 endosomes); see Supplementary Methods for the estimation of the Sara levels at the endosomes), while endosomes with lower levels of Sara do not preferentially move towards the central spindle (Fig. 1e,f). Since the amount of Sara in endosomes forecasts their targeting to the cleavage plane, Sara itself could in principle play a role in endosomal targeting to the central spindle. These results, although limited by the fact that they were obtained in Sara overexpression conditions, prompted us to look at Sara loss of function mutants and to systematically study the dynamics of Sara endosomes at endogenous Sara levels by looking at iDelta^20′^.

### Sara itself controls endosomal behaviour

**Figure 2:**
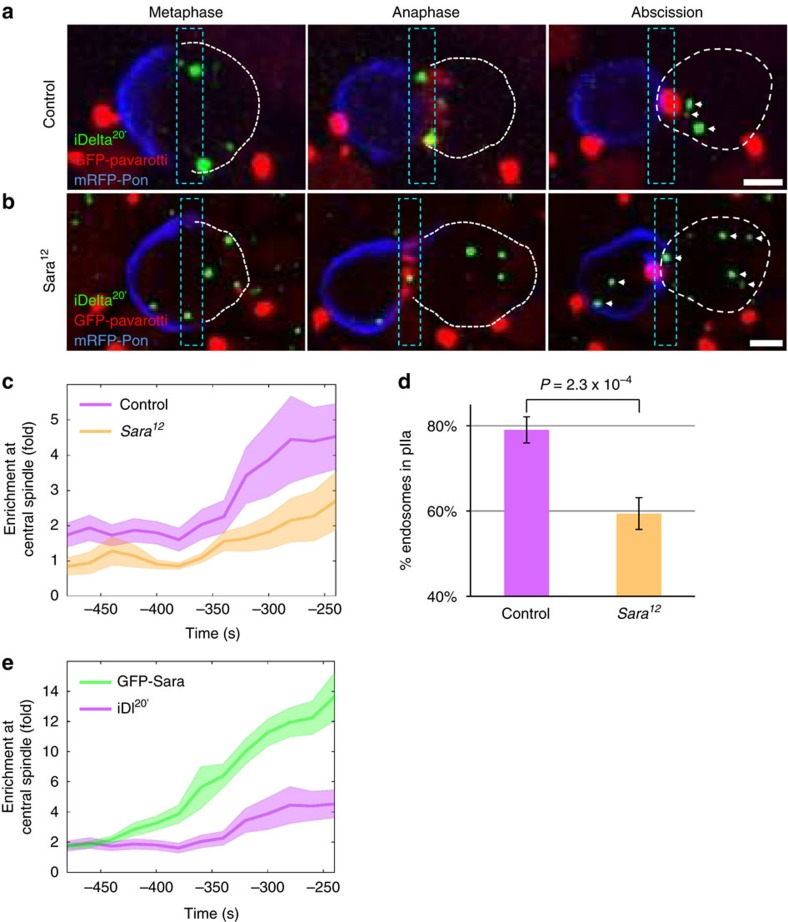
Sara contributes to central spindle targeting and asymmetric segregation of endosomes. (**a**,**b**) Images from time-lapse movies (*z*-projections) of dividing wild type (**a**) and *Sara*^*12*^ mutant SOPs (**b**) in metaphase, anaphase and at abscission. Arrowheads indicate the position of endosomes. Note that the GFP-Pavarotti signal has been displayed in the red channel. Scale bar, 3 μm. (**c**) Enrichment of iDelta^20′^ endosomes in the central spindle area in control conditions (purple; *n*=17–35, *N*=9) and in *Sara*^*12*^ mutants (orange; *n*=9–23, *N*=8). (**d**) Percentage of iDelta^20′^ endosomes segregating to the pIIa daughter cell upon division of WT (*n*=23, *N*=6) and *Sara*^*12*^ SOPs (*n*=20, *N*=7). (**e**) Enrichment of iDelta^20′^ endosomes (purple; *n*=17–35, *N*=9) and GFP-Sara endosomes (green; *n*=12–26, *N*=5). All error bars represent s.e.m.

To address a potential role of Sara on central spindle targeting and asymmetric segregation, we tracked and quantified the behaviour of the endosomes in a Sara loss of function mutant (*Sara*^*12*^) and in conditions of Sara overexpression in the SOP (*Neur-Gal4; UAS-GFP-Sara*). In *Sara*^*12*^ SOPs, targeting of iDelta^20′^ endosomes to the cleavage plane is severely impaired (Fig. 2a–c for iDelta^20′^ uptake assay and Supplementary Fig. 4 for endogenous Delta). Consistent with the fact that the asymmetric dispatch of endosomes to pIIa requires first their targeting to the central spindle as previously shown^15^, in *Sara*^*12*^ SOPs the dispatch to the pIIa daughter is strongly affected (Fig. 2b,d; Supplementary Fig. 4). A slight bias (60% pIIa targeting) is, however, retained in the mutant, consistent with a previous report^10^.

**Figure 3:**
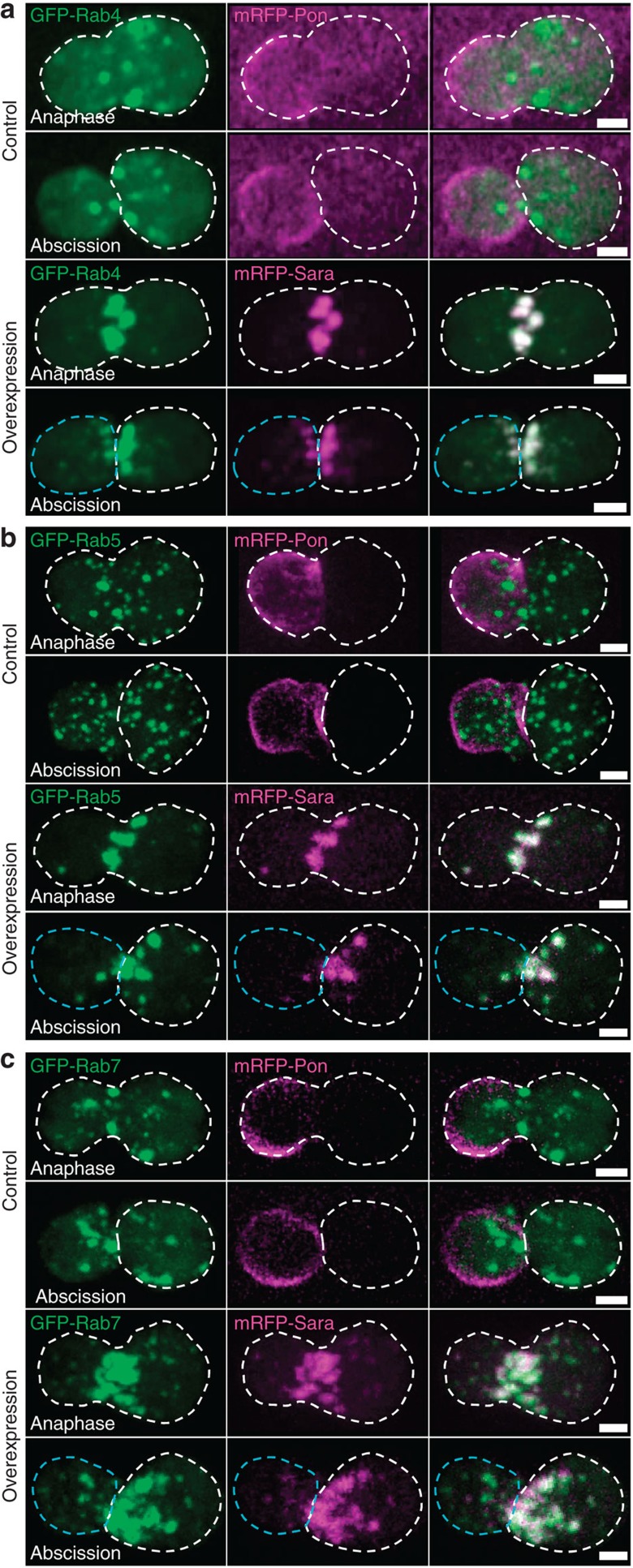
Sara overexpression increases targeting to the central spindle of Rab4, Rab5 and Rab7 endosomes. (**a**–**c**) Images from time-lapse movies (*z*-projections) of SOPs in anaphase and at abscission expressing GFP-Rab4 (**a**), GFP-Rab5 (**b**) or GFP-Rab7 (**c**) together with mRFP-Pon (upper panels) or with mRFP-Sara overexpression (lower panels). The dashed white line represents the cell outline; anterior is on the left. Scale bar, 3 μm.

Conversely, overexpression of Sara increases targeting to the central spindle (Fig. 2e). In these conditions, Sara is found not only in Rab5 endosomes, but also in Rab7 late endosomes as well as in the Rab4 recycling endosomes (Fig. 3a–c; Supplementary Fig. 3a–c). Correlating with this, Rab4, Rab5 and Rab7 endosomes, which are not all recruited to the central spindle in wild-type conditions, are now targeted to the central spindle upon Sara overexpression and are asymmetrically targeted (Fig. 3a–c).

Furthermore, consistent with the correlation that we observed between the levels of Sara at the endosomes and their displacement towards the cleavage plane (Fig. 1e,f), quantification of central spindle targeting of the Sara endosomes upon its overexpression shows that targeting of the endosomes to the cleavage plane is increased by a factor of 2.5 in these conditions (Fig. 2e). These observations indicate that Sara plays a crucial role on the targeting of the endosomes to the spindle and the subsequent dispatch of the Notch/Delta containing endosomes to pIIa. Does this play a role during Notch-dependent asymmetric cell fate assignation?

### Sara contributes to Notch asymmetric signalling

**Figure 4:**
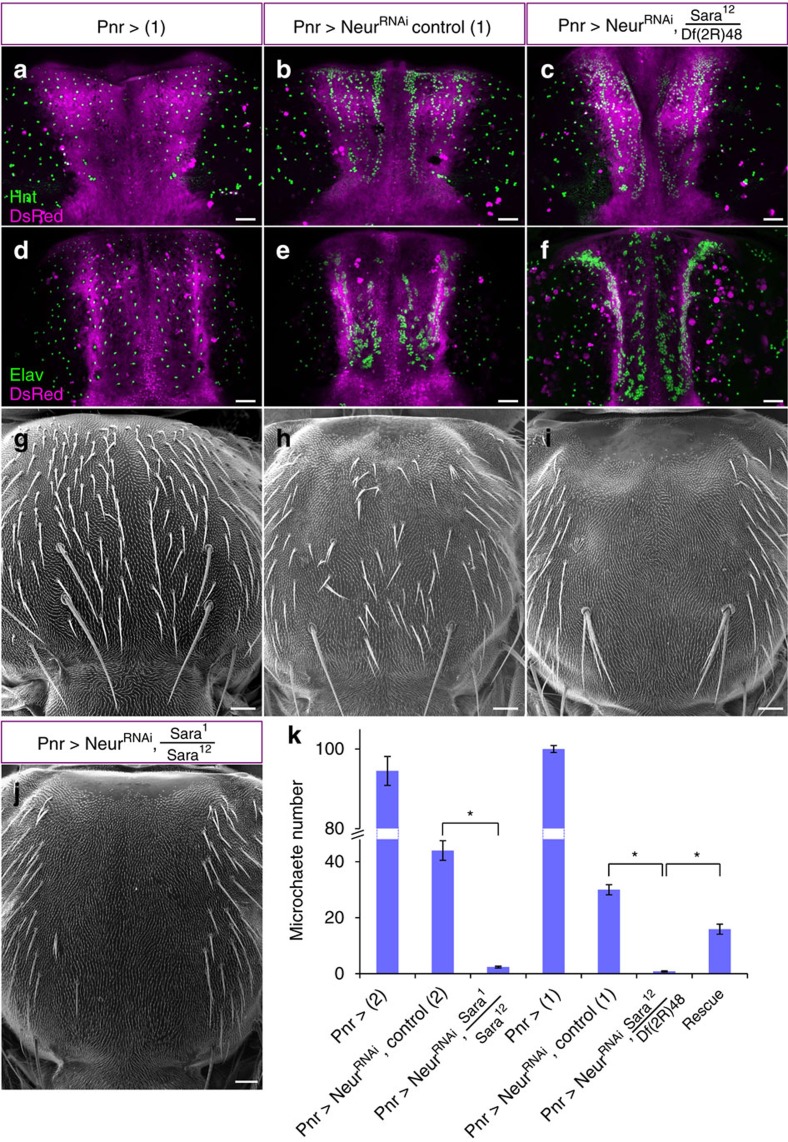
Sara contributes to Notch-dependent asymmetric cell fate assignation. (**a**–**f**) Immuno-stainings (*z*-projections) of nota for the SOP marker Hindsight (**a**–**c**) and the neuron marker Elav (**d**–**f**) of ‘Pnr>' controls (**a**,**d**), ‘Pnr>Neur^RNAi^' controls (**b**,**e**) and ‘Pnr>Neur^RNAi^, Sara^12^/Df(2 R)48′ *Sara*^*-*^ mutant animals (**c**,**f**). Scale bar, 50 μm. (**g**–**j**) Scanning electron micrographs of the same genotypes as in **a**–**f** (**g**–**i**) and Pnr>*Neur*^*RNAi*^, *Sara*^*1*^/*Sara*^*12*^ (**j**). Scale bars, 50 μm. (**k**) Number of microchaete present in the Panier (Pnr) expression domain in the different genotypes. Asterisks indicate *P*-values <0.05 (see Supplementary Methods for details and genotypes). All error bars represent s.e.m.

Sara function contributes to cell fate assignation through asymmetric Notch signalling, but this activity is redundantly covered by Neuralized (Fig. 4). Neuralized E3 Ubiquitin ligase does play an essential role during the endocytosis and activation of the Notch ligand Delta^6^. Therefore, during larval development, Neuralized is essential for Notch-mediated lateral inhibition in the proneural clusters, which leads to the singling-out of SOP cells from the proneural clusters^16^. Later, during pupal development, Neuralized appears as a cortical crescent in the pIIb side of the dividing SOPs, thereby biasing Delta activation in the pIIb cell and asymmetric activation of Notch in pIIa^6^.

Consistently, a partial loss of function of Neuralized by RNAi interference in the centre of the notum (*Pnr>NeurRNAi* Control) showed lateral inhibition defects in the proneural clusters, causing the appearance of supernumerary SOPs (Fig. 4a,b) as well as asymmetric Notch signalling defects in the SOP lineage, leading to supernumerary neurons and loss of the external shaft/socket cells in the lineage (Fig. 4d,e,g,h)^15^. The remaining Neuralized activity in this partial loss of function condition allows many sensory organs (more than forty in the centre of the notum) to perform asymmetric cell fate assignation and to develop, as in wild type, into structures containing at least the two external cells (shaft and socket; Fig. 4g,h,k).

In *Pnr>Neur*^*RNAi*^*, Sara*^*12*^*/Df(2R)48* transheterozygote mutants, the number of supernumerary SOPs is increased by 35% with respect to the P*nr>NeurRNAi* controls (668±38 versus 498±52; Fig. 4b,c; see also Supplementary Methods ‘Quantification of the Neur phenotypes'). This indicates that during lateral inhibition, Sara endosomes contributes to Notch signalling. This general role of Sara is uncovered when the Neuralized activity during Notch signalling is compromised.

In the case of Neuralized, its localization to the anterior cortex biases Notch signalling to be elicited in the pIIa cell. This is the same in the case of Sara endosomes: asymmetric dispatch of Sara endosomes also biases Notch signalling to pIIa^10^. Indeed, in *Pnr>Neur*^*RNAi*^*, Sara*^*12*^*/Df(2R)48* transheterozygote mutants, the number of bristles (external shaft/socket cells) in the notum is strongly reduced at the expense of supernumerary neurons compared to the *Pnr>NeurRNAi* controls (Fig. 4d–i,k). This indicates that Notch-dependent asymmetric cell fate assignation in the SOP lineage is synergistically affected in the Sara/Neuralized mutant. This implies that the SOP lineages which still could generate bristles with lower levels of Neuralized function in *Pnr>Neur*^*RNAi*^ need Sara function to perform asymmetric cell fate assignation: in *Pnr>Neur*^*RNAi*^, *Sara*^*12*^*/Df(2R)48* and *Pnr>Neur*^*RNAi*^, *Sara*^*12*^*/Sara*^*1*^ transheterozygote mutants, these lineages failed to perform asymmetric signalling, causing the notum to be largely bald (Fig. 4g–k). Therefore, Sara contributes to Notch signalling and asymmetric cell fate assignation, as observed in conditions in which other redundant systems for asymmetric Notch signalling are compromised.

Both Neuralized and Sara play general roles in Notch signalling: they are both involved in lateral inhibition at early stages and, at later stages, in asymmetric cell fate assignation. Indeed, both *Neuralized* and *Sara* mutants show early defects in lateral inhibition and, accordingly, they show supernumerary SOPs (Fig. 4b,c). In addition, *Neuralized* and *Sara* mutant conditions also show defective Notch signalling during cell fate assignation in the SOP lineage and therefore cause the transformation of the cells in the lineage into neurons (Fig. 4d–k). In this later step, Notch signalling is asymmetric. The possibility that both Sara and Neuralized play key roles in ensuring the asymmetric nature of this signalling event is only correlative: in the case of Neuralized, it is enriched in the anterior cortex of the cell, which will give rise to pIIb^6^; in the case of Sara, (i) both Delta and Notch are cargo of these endosomes, (ii) cleaved Notch is seen in the pIIa endosomes and (iii) Sara endosomes are dispatched asymmetrically to pIIa^10^. It is tantalizing to conclude that the asymmetric localization of these two proteins mediate the asymmetric nature of Notch signalling in the SOP lineage, but further assays will be necessary to unambiguously address this issue. Clonal analysis^6^ is unfortunately a too slow assay to sort out the specific requirement of these cytosolic factors (Sara and Neuralized) in the pIIa versus the pIIb cell (see ‘Quantification of the Neur phenotypes' in Supplementary Methods).

### Sara binds PP1c and its Sds22 regulatory subunit

Sara mediates the targeting of Notch/Delta containing endosomes to the central spindle and could contributes to Notch-mediated asymmetric signalling in the SOP lineage. What machinery controls in turn the Sara-dependent targeting of endosomes to the central spindle?

**Figure 5:**
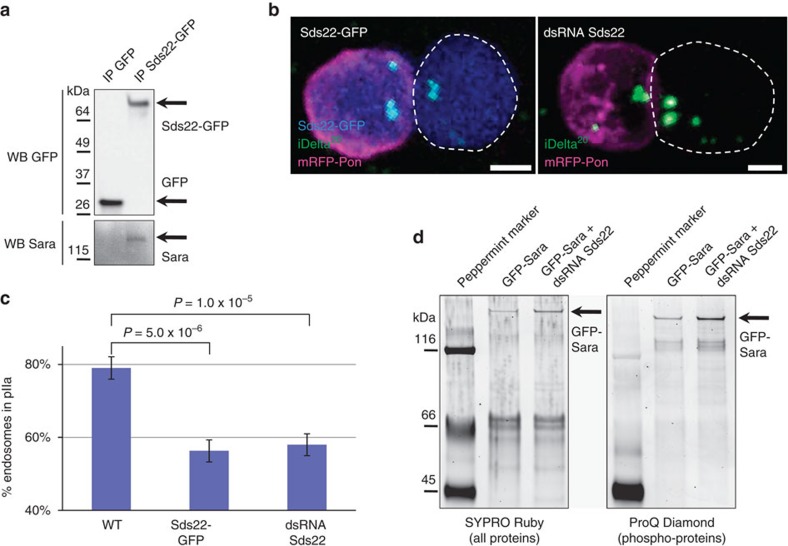
Sara binding to Sds22 and phosphorylation-dependent dynamics of Sara endosomes. (**a**) Sara co-immunoprecipitates with Sds22-GFP (second lane), but not with GFP (first lane). (**b**) Images from time-lapse movies (*z*-projections) of SOPs expressing Sds22-GFP (left) or a dsRNA against Sds22 (right) at abscission. Scale bar, 2 μm. (**c**) Percentage of iDelta^20′^ endosomes segregating to the pIIa daughter cell upon division of WT SOPs (*n*=23 cells in *N*=6 animals) and SOPs expressing Sds22-GFP (*n*=35, *N*=6) or a dsRNA against Sds22 (*n*=21, *N*=3). Error bars represent s.e.m. (**d**) SDS–PAGE followed by stainings of all proteins (SYPRO Ruby; left) and of phosphoproteins (ProQ Diamond; right) reveals a 40%-increase (normalized to the total Sara protein quantity) in the level of phospho-GFP-Sara upon inhibition of Sds22.

Previous proteomic studies uncovered *bona fide* Sara-binding factors, including the Activin pathway R-Smad, Smox^17^ and the beta subunit of the PP1c serine-threonine phosphatase (PP1β(9C)^18^. In an IP/Mass Spectrometry approach, we confirmed those interactions and found in addition to PP1β(9C), two of the other three Drosophila isoforms of PP1c: PP1α(87B) and PP1α(96A) (See Methods). Furthermore, we also found the PP1c regulatory subunit Sds22 (ref. 19), suggesting that Sara binds the full serine-threonine PP1 phosphatase complex. We confirmed the interaction with Sds22 by immunoprecipitation of overexpressed Sds22-GFP and western blot detection of endogenous Sara in the immunoprecipitate (Fig. 5a).

### Sds22-dependent asymmetric dispatch of Sara endosomes

Prompted by these results, we then explored whether the PP1 complex plays a role in the asymmetric targeting of the Sara endosomes by manipulating the activity of Sds22, the common regulatory unit in all the complexes containing the different PP1 isoforms^19^. We overexpressed Sds22 specifically during SOP mitosis, by driving Sds22-GFP under the Neur-Gal4 driver with temporal control by the Gal80^ts^ system^19^. Figure 5b and c shows that, in SOPs where PP1-dependent dephosphorylation is enhanced by overexpressing Sds22, the Sara endosomes fail to be dispatched asymmetrically toward the pIIa daughter cell.

We then further looked at the role of PP1-dependent dephosphorylation in the SOP, by knocking down Sds22 (through a validated Sds22-RNAi) as described above for the overexpression assay. Loss of function Sds22 did also affect the asymmetric targeting of endosomes (Fig. 5b,c). These data uncover a key role for phosphorylation and PP1-dependent dephosphorylation as a switch that contributes to the asymmetric targeting of Sara during asymmetric cell division.

The observations raise the question of which is the step in the asymmetric dispatch of the endosomes that is controlled by the levels of phosphorylation: central spindle targeting, central spindle detachment or targeting to the pIIa cell? PP1/Sds22-dependent dephosphorylation controls a plethora of mitotic events, including mitotic spindle morphogenesis^20^, cortical relaxation in anaphase^20^,^21^, epithelial polarity and cell shape^19^, Aurora B activity and kinetochore–microtubule interactions^22^,^23^ as well as metabolism, protein synthesis, ion pumps and channels^24^. Therefore, to establish the specific event during the asymmetric dispatch of Sara endosomes that is controlled by PP1/Sds22 dephosphorylation, we first focused on the phosphorylation state of Sara itself and its previously identified phosphorylation sites^18^. This allowed us to specifically interfere with this phosphorylation event and thereby untangle it from other cellular events also affected by dephosphorylation.

### Sara phosphorylation state controls targeting and departure

We showed that PP1/Sds22 binds Sara (Fig. 5a). It has previously been shown that mammalian Sara itself is phosphorylated at multiple sites^17^ and that the level of this Sara phosphorylation is independent on the level of TGF-beta signalling^17^. Three phosphorylation sites have been identified^25^ at position S636, at position S709, and at position S774 in Sara protein and we confirmed these sites by Mass Spectrometry of larval tissue expressing GFP-Sara. Phosphorylation of Sara had been previously reported to be implicated in BMP signalling during wing development^18^,^25^. However, the role of these three phosphorylation sites during asymmetric division are to date unknown.

We confirmed by ProQ-Diamond phospho-staining of immunoprecipitated GFP-Sara that Sara is phosphorylated (Fig. 5d). To test whether PP1/Sds22 controls the phosphorylation state of Sara, we performed ProQ-Diamond stainings of GFP-Sara with and without down-regulation of Sds22. Downregulating Sds22 induced a 40%-increase in the normalized quantity of phosphorylated Sara (Fig. 5d), showing that PP1/Sds22 does control the phosphorylation state of Sara.

**Figure 6 f6:**
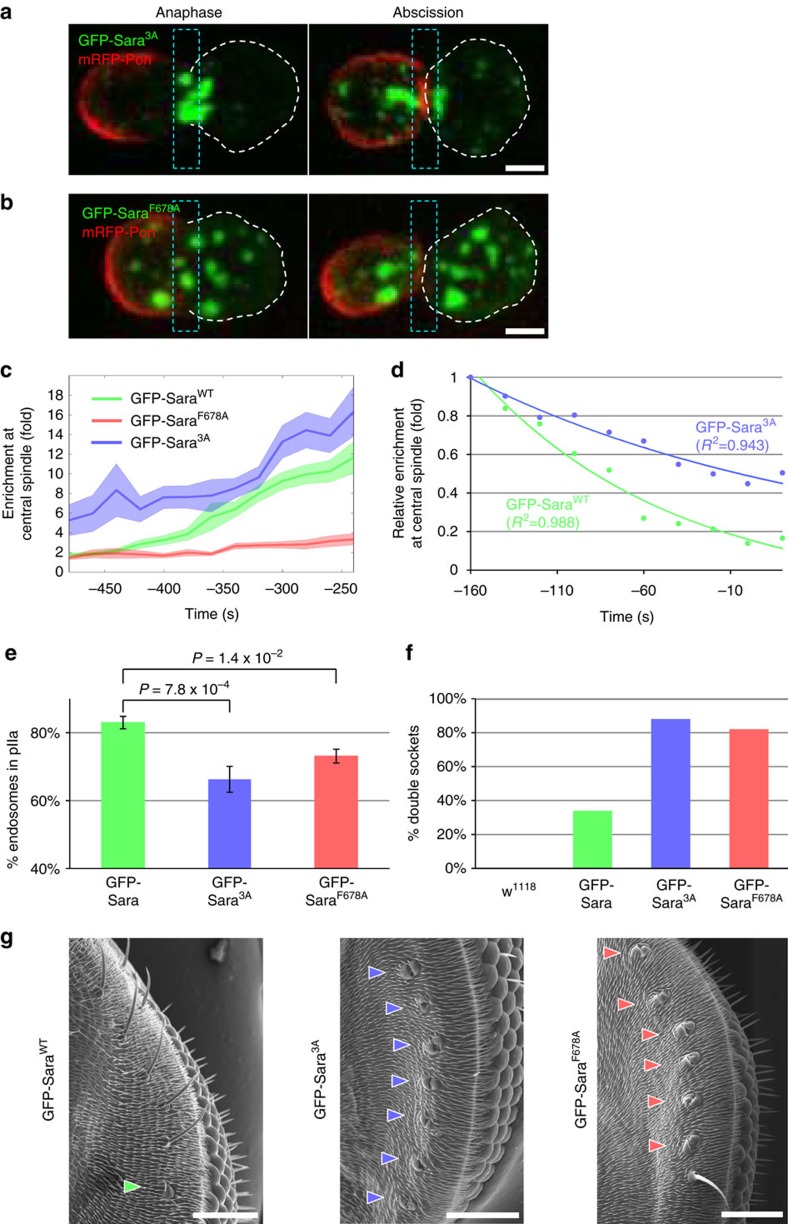
The state of phosphorylation of Sara controls the targeting and the departure of endosomes from the central spindle. (**a**,**b**) Images from time-lapse movies (*z*-projections) of dividing SOPs expressing GFP-*Sara*^*3A*^ (**a**) or GFP-*Sara*^*F678A*^ (**b**) in anaphase and at abscission. Scale bar, 3 μm. (**c**) Enrichment of Sara endosomes in the central spindle area upon expression of GFP-Sara (green; *n*=12–26, *N*=5), GFP-*Sara*^*3A*^ (blue; *n*=9–22, *N*=7) and GFP-*Sara*^*F678A*^ (red; *n*=6–24, *N*=7). (**d**) Relative enrichments of Sara endosomes (normalized to their respective maximal values) from two representative cells expressing GFP-Sara (green) and GFP-*Sara*^*3A*^ (blue) measured in the 2 μm-wide central spindle area; dots represent data points, and the lines represent the exponential fits used to estimate the residence times. (**e**) Percentage of Sara endosomes segregating to the pIIa daughter cell upon division of SOPs expressing GFP-Sara (green; *n*=24, *N*=4), GFP-*Sara*^*3A*^ (blue; *n*=24, *N*=4) or GFP-Sara^F678A^ (red; *n*=24, *N*=7). (**f**) Percentage of double sockets present in the post-orbital region upon expression (*Neur-Gal4, tub-Gal80*^*ts*^) of GFP-Sara (green; *n*=96 lineage events in *N*=16 eyes from pharate adults), GFP-*Sara*^*3A*^ (blue; *n*=76 lineage events in *N*=14 eyes from pharate adults) and GFP-*Sara*^*F678A*^ (red; *n*=67 lineage events in *N*=8 adult eyes) or in control white pupae (*n*=74 events in *N*=16 adult eyes). (**g**) Scanning electron micrographs of the post-orbital region of GFP-Sara, GFP-*Sara*^*3A*^ and GFP-*Sara*^*F678A*^ expressing animals. Arrowheads, double sockets. Scale bar, 50 μm. All error bars represent s.e.m.

To study the role of Sara phosphorylation during asymmetric targeting of the endosomes, we analysed the mitotic behaviour of the endosomes in conditions of overexpression of mutant versions of Sara where (i) the three phosphorylated Serines (at position S636, S709, and S774) were substituted by Alanine (phosphorylation defective: GFP-*Sara*^*3A*^) or (ii) the PP1 interaction was abolished by an F678A missense mutation in the PP1 binding domain (hyper-phosphorylated: GFP-*Sara*^*F678A*^)^18^. We controlled that neither mutation affects the general levels of abundance of the Sara protein in SOPs (Supplementary Fig. 2a), the targeting of Sara itself to the endosomes (Fig. 6a,b), nor the residence time of Sara in endosomes as determined by FRAP experiments (Supplementary Fig. 2b–e). Also, the targeting dynamics of internalized Delta to endosomes are not affected in these mutants (20 min after internalization, iDelta^20′^ is found in endosomes; Supplementary Fig. 6a,b), consistent with the fact that these mutant endosomes are still functional during Notch signalling (see below).

Upon overexpression of GFP-*Sara*^*3A*^ in SOPs, the rate of targeting of the endosomes to the central spindle is greatly increased (Fig. 6a,c). In addition, GFP-*Sara*^*3A*^ shows impaired departure from the spindle: while the residence time of Sara endosomes at the central spindle after their recruitment is around 100 s in wild type (see above), GFP-*Sara*^*3A*^ endosomes stay at the spindle significantly longer (151±21 s; *n*=15 cells; see Fig. 6d). In GFP-*Sara*^*3A*^ endosomes, impaired departure leads to defective asymmetric targeting to the pIIa cell (Fig. 6a,b,e; Supplementary Fig. 6B) while, in *wild type*, departure from the central spindle occurs well before abscission, in the GFP-*Sara*^*3A*^ condition, endosomes that did not depart are caught at the spindle while abscission occurs. These data indicate that the endosomal targeting to the central spindle is greatly favoured when these three sites in Sara are dephosphorylated and suggest that the departure from the microtubules of the central spindle requires that the endosomes are disengaged by phosphorylation of Sara.

Loss of Sara phosphorylation in these sites impairs disengagement from the central spindle. Conversely, impairing Sara binding to the PP1 phosphatase results in defective targeting to the central spindle. Indeed, when binding of Sara to the PP1/Sds22 phosphatase is impaired in the GFP-*Sara*^*F678A*^ overexpressing SOP mutants, Sara endosomes fail to be targeted to the spindle (Fig. 6b,c). Mistargeted away from the central spindle, the GFP-*Sara*^*F678A*^ endosomes fail thereby to be asymmetrically targeted to the pIIa cell (Fig. 6e; Supplementary Fig. 6B). Loss and gain of function phenotypes of the Phosphatase regulator Sds22 during endosomal spindle targeting (Supplementary Fig. 5) support the role of Sara phosphorylation during targeting to the central spindle microtubules suggested by the GFP-*Sara*^*3A*^ and GFP-*Sara*^*F678A*^ experiments.

What are the functional consequences on signalling of impaired phosphorylation/dephosphorylation in Sara mutants? The presence of Sara in endosomes is itself essential for Notch signalling (Fig. 4). Sara loss of function mutants show a phenotype in SOP specification (supernumerary SOPs) as well as during fate determination within the SOP lineage (all cells in the lineage acquire a neural fate). In addition, we show that Sara is also essential for the targeting of endosomes to the spindle (Fig. 2): in the absence of Sara, endosomes fail to move to the spindle in the SOP. They are therefore dispatched symmetrically, but those endosomes do not mediate Notch signalling. As a consequence, both daughters fail to perform Notch signalling in sensitized conditions in which Neuralized is compromised. The result is a Notch loss of function phenotype: the whole lineage differentiates into neurons.

In both *Sara*^*3A*^ and *Sara*^*F678A*^ mutants, because of reasons that are different in the two cases (either they do not go to the spindle or their departure from the spindle is impaired), functional Sara endosomes are dispatched symmetrically (Fig. 6a,b,e). In contrast to the situation in the Sara loss of function mutant, those endosomes are functional Sara signalling endosomes, which can mediate Notch signalling in both cells. Consistently, Fig. 6f,g shows that these mutations therefore cause a gain of function Sara signalling phenotype: supernumerary sockets are seen in the lineages (88% of the lineages for *Sara*^*3A*^ and 82% of the lineages for *Sara*^*F678A*^). A milder version of this phenotype can be also seen by overexpressing *wild-type* Sara (34% of the lineages) consistent again with some gain of function Notch signalling phenotype when Sara concentrations are elevated. In summary, this implies that the 3A and F678A mutations impair the phosphorylation state of Sara (with consequences in targeting), but not its function in Notch signalling.

These results indicate that Sara itself plays a key, rate limiting role on the asymmetric targeting of the endosomes (summarized in Supplementary Fig. 7) by controlling the targeting to the spindle (Figs 1e,f and 2c) and its departure (Fig. 2d). Maturation of the early endosomes by accumulating PI(3)P leads to accumulation of the PI(3)P-binding protein Sara to this vesicular compartment. At the endosome, the phosphorylation state of Sara indeed determines central spindle targeting (Fig. 6a-c) and departure (Fig. 6d): in its default, dephosphorylated state, Sara is essential to engage the endosomes with the mitotic spindle. Phosphorylation of Sara disengages the endosomes from the central spindle allowing the asymmetric departure into the pIIa cell.

## Methods

All experimental procedures are described in detail in the Supplementary Methods section.

### Fly lines and fly handling

The fly handling conditions, the list of the fly stocks used and the list of all genotypes can be found in the Supplementary Methods section. For the procedures to generate the UAS-GFP-*Sara*^*3A*^ and UAS-GFP-*Sara*^*F678A*^ transgenic fly lines, see below.

### DNA constructs

The *Sara*^*3A*^ mutation consists in the replacement in the Sara protein of S^636^, S^709^ and S^774^ by alanines; introduction of theses mutations into the Sara sequence was done by Genscript UAS Inc. The *Sara*^*3A*^ construct, GFP tagged in 5′ was inserted into a pUAST vector (DGRC) leading to an N-terminal EGFP fusion. The GFP-*Sara*^*F678A*^ construct was amplified directly from *UAS-Sara*^*F678A*^ flies^18^, GFP tagged in 5′ and cloned into pUAST vector (DGRC).

All injections into fly embryos were done by BestGene Inc.

### SDS–PAGE/western blots

Protein extracts were loaded on Nupage Bis Tris 4–12% gradient gels. The gels were transferred to PVDF membranes in the following transfer buffer: 25 mM Tris, 192 mM glycine; 20% methanol; pH 7.5. After the transfer, the membrane was rehydrated in distilled water and blocked during 30 min in PBT (PBS, 0.1% Tween-20, pH 7.5) with 5% non-fat dried milk. Primary antibodies, anti-Sara antibodies^10^ from rabbit and anti-GFP antibodies from mouse (Santa Cruz # sc-8334), were diluted at 1 μg ml^−1^ in blocking solution, and incubated overnight at 4 °C. The membrane was then washed 3 × 5 min in PBT, HRP-coupled secondary antibodies (Jackson Immunoresearch 1:10,000 dilution) were diluted in blocking solution and incubated during 1 h at room temperature. The Benchmark Prestained Protein Ladder (Thermo Scientific # 10748-010) was used to monitoring protein sizes. Finally, western blots were revealed using Super Signal West Pico Chemiluminescent Substrate (Thermo Scientific # 34080) and a Vilber Lourmat Fusion imager (Supplementary Fig. 8).

### Phosphorylation stainings

ProQ Diamond phosphoprotein fluorescent gel stain (Molecular Probes # 33300) and SYPRO Ruby protein fluorescent gel stain (Molecular Probes # M33305) were used to quantitatively label the immunoprecipitated proteins and measure their phosphorylation state according to the instructions of the manufacturer (Supplementary Fig. 9). The PeppermintStick phosphoprotein molecular weight standards (Molecular Probes # P27167) were used as a control. Detection was done with the Ettan DIGE Imager (GE Healthcare).

### Immuno-precipitations and mass spectrometry

Brains and imaginal discs of 100 third instar larvae were dissected and squashed into 500 μl of lysis buffer: 50 mM Tris; 150 mM NaCl; 50 mM Sucrose; 5 mM EDTA; 5 mM ATP; 1 mM DTT; 0.3% Triton X-100; pH 7.5 and protease inhibitors (Complete mini tablets Roche # 05892791001). The extract was then incubated 40 min at 4 °C on a rotating wheel, and cellular debris were cleared by centrifugation (16,000*g*, 10 min at 4 °C). Twenty-five microlitre of GFP-Trap beads slurry (Chromotek # 090703001A) were equilibrated with Triton-free lysis buffer and incubated with the cleared extract. Immunoprecipitation was performed during 2 h at 4 °C with mild agitation. Beads were then washed three times with lysis buffer, two times with Triton-free lysis buffer and finally resuspended in 40 μl Laemmli sample buffer. Samples were further processed for SDS–PAGE and western blot.

For mass spectrometry, immunoprecipitates were processed by SDS–PAGE on 10% Precise Precast Protein Gels (Thermo Scientific). Bands were cut from the gel, and further processed for mass spectrometry using the NanoLC-ESI-MS/MS technique.

### Live notum and pupae imaging

Live pupae imaging in Figs 1 and 2; 3b,c and 5; and 6, [Fig f6] was performed as followed^1^: Double-sided scotch tape was glued to a microscopy slide, and a pupa was gently deposited on it. The operculum was removed by pulling on a spiracle, and the part of the pupal case covering the thorax was gently torn and removed. Spacers composed of four coverslips glued together were then stuck on each side of the pupa and finally, a small drop of Voltalef 10S oil was deposited on a glass coverslip to cover the pupa for imaging. Nail polish was used to glue the coverslip to the underlying spacers.

Live fly notum imaging in Fig. 3a, Supplementary Figs 2, 3, 5 and 6 was performed as followed^1^: imaging was performed on dissected fly nota, fly notum dissection and SOP imaging was performed in clone 8 media after embedding into a fibrinogen clot^13^to diminish tissue movements during 3D image acquisition.

Live imaging was performed on a 3i Marianas confocal spinning-disc microscope (Intelligent Imaging Innovations) equipped with a × 63 Oil numerical aperture (NA) 1.4 objective, a CSUX-M1 spinning-disc head (Yokogawa) and an Evolve EM-CCD camera (Photometrics), as described in (ref. 1). Consecutive *z* stacks were acquired every 10, 12 or 20 s and maximum intensity *z*-projections are shown.

### Antibody uptake assays

Fluorescent Delta antibody uptake to label the Sara endosomes was performed with a 5-min pulse (3.4 μg ml^−1^ of labelled Delta antibody in clone 8 or 0.7 μm ml^−1^ of the Delta and Zenon mix) and a 20-min chase (referred to as iDelta^20′^). Anti-Delta antibodies (C594.9B, Developmental Studies Hybridoma Bank) were either coupled to fluorescent Zenon secondary antibodies (Invitrogen Z-25008) or covalently coupled to Atto647N (ref. 15; Atto tech). Uptake assays were performed in *ex vivo* fly nota in a fibrinogen clot^1^,^10^,^13^ (Figs 2a–e and 5b,c; Supplementary Fig. 6).

### Immunofluorescence

For lineage staining (Fig. 4a–f), fly nota were dissected 12 h (for Hindsight staining) or 27.5 h (for Elav staining) after puparium formation and processed for immunofluorescence as described^10^ using either rat anti-Elav at 22 μg ml^−1^ (clone 7E8A10, Developmental Studies Hybridoma Bank) or mouse anti-Hindsight at 4.44 μg ml^−1^ (clone 1G9, Developmental Studies Hybridoma Bank) followed by anti-rat Cy5-coupled secondary antibodies (Biozol) or anti-mouse Alexa 647-coupled secondary antibodies (Thermo Fischer) at a 1/100 dilution. Dissected fly nota were then embedded in a fibrinogen clot^1^ and imaged in PEM. Image acquisition was performed on an Olympus FV1000 confocal scanning microscope using a × 60 NA 1.42 oil objective, acquiring voxels of 621 × 621 × 1,000 nm^3^. Images correspond to maximum intensity *z*-projections.

For immunostaining of endogenous Delta in *Sara*^*1*^*/Sara*^*12*^ mutant SOPs, *w*^*1118*^*; Sara*^*1*^ */ Sara*^*12*^ pupae were dissected 12 h after puparium formation and processed for immunofluorescence as described^10^ in a three separated incubation times: a mouse anti-Hindsight (4.44 μg ml^−1^; clone 1G9, Developmental Studies Hybridoma Bank) then by highly-crossed-adsorbed anti-mouse Alexa 488-coupled secondary antibodies (Thermo Fischer) at a 1/50 dilution and followed by a Mouse anti-Delta monoclonal antibodies at 3.4 μg ml^−1^ (initially from C594.9B, Developmental Studies Hybridoma Bank) labelled by Atto647N (ref. 15). Coverslips were mounted in Prolong Gold anti-fade reagent.

### Image processing and statistical analysis

Detection of endosomes was performed as previously described^26^, and trajectories of endosomes were reconstructed by connecting the measured positions using a modified Vogel-algorithm^26^. For a complete description of the image analysis and statistical analysis procedures, see the see Supplementary Methods section.

### Data availability statement

Materials, codes and associated protocols of this study are available on request from the corresponding author (M.G.-G.).

## Additional information

**How to cite this article:** Loubéry, S., Daeden, A. *et al*. Sara phosphorylation state controls the dispatch of endosomes from the central spindle during asymmetric division. *Nat. Commun.* **8,** 15285 doi: 10.1038/ncomms15285 (2017).

**Publisher's note:** Springer Nature remains neutral with regard to jurisdictional claims in published maps and institutional affiliations.

## Supplementary Material

Supplementary Information Supplementary Figures, Supplementary Methods and Supplementary References

Supplementary Movie 1 Tracking of Sara endosomes in dividing SOPs. The movie corresponds to the cell shown in Fig. 1A. GFP-Sara is represented in gray levels, the red line represents the Pon crescent limit as detected by the algorithm and for clarity purposes, only a few representative tracks of endosomes are shown in different colours.
