# Supplementary material for: Sara phosphorylation state controls the dispatch of endosomes from the central spindle during asymmetric division
Source: Nat Commun. 2017 Jun 6;8:15285. doi: 10.1038/ncomms15285 (PMC5467175; doi:10.1038/ncomms15285)
Supplement: Supplementary Information — Supplementary Figures, Supplementary Methods and Supplementary References [file ncomms15285-s1.pdf]

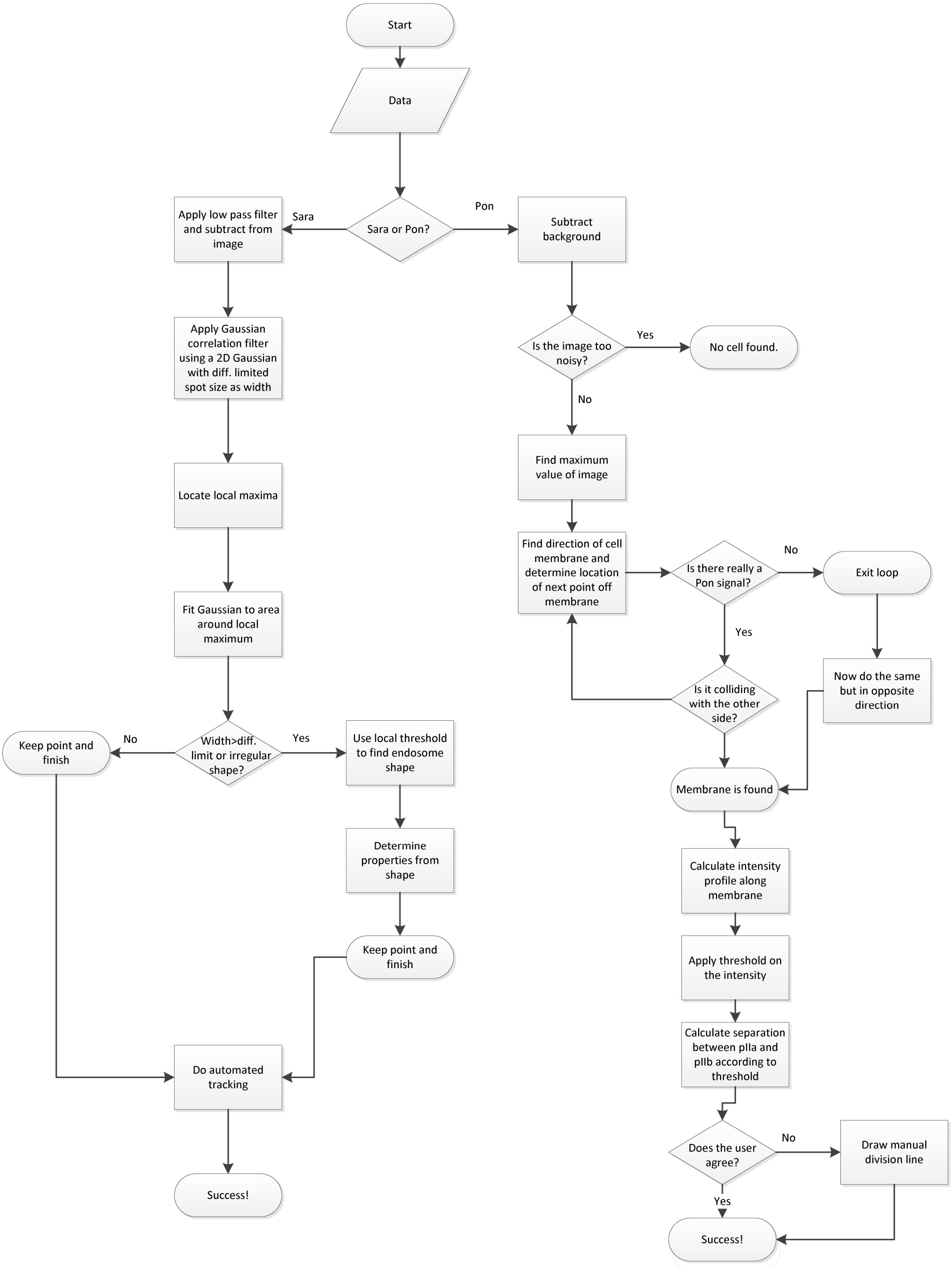

Supplementary Figure 1. Flowchart of the 3D endosome tracking and Pon crescent detection software.

Supplementary Figure 1. Flowchart of the 3D endosome tracking and Pon crescent detection software.

For details of the detection and tracking procedure, see the Supplementary Methods section.

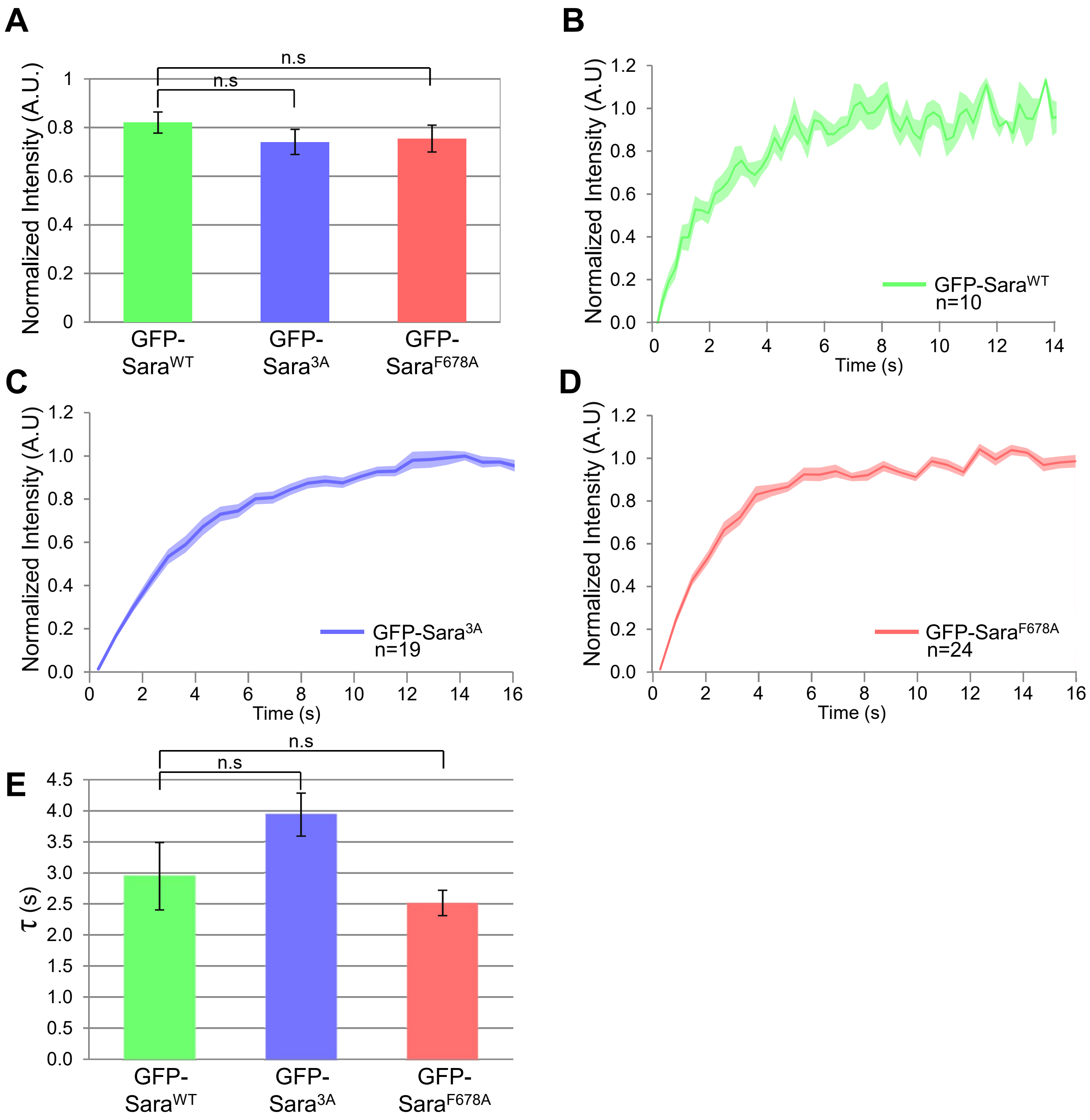

Supplementary Figure 2. Expression of GFP-Sara , GFP-*Sara*^{3A} or GFP-*Sara*^{F678A} does not change expression levels and the stability of Sara proteins on endosomes after Fluorescence Recovery After Photobleaching.

Supplementary Figure 2. Expression of GFP-Sara, GFP-*Sara*^{3A} or GFP-*Sara*^{F678A} does not change expression levels and the stability of Sara proteins on endosomes after Fluorescence Recovery After Photobleaching.

(A) Total fluorescence intensity of SOPs expressing GFP-Sara (green; n=17 SOPs), GFP-*Sara*^{3A} (blue; n=24) and GFP-*Sara*^{F678A} (red; n=16) normalized by the Pon signal, in metaphase, showing that there is no difference levels of expression (ANOVA test, P>0.5, n.s.).

(B-D) Fluorescence intensity curve after FRAP of single endosome of GFP-Sara (green; n=10), GFP-*Sara*^{3A} (blue; n=19) and GFP-*Sara*^{F678A} (red; n=24) expressing SOPs. The shaded area represents the standard error of the mean.

(E) Average Residence time τ (s) in endosomes of GFP-Sara (green; n=10), GFP-*Sara*^{3A} (blue; n=19; Mann-Whitney Rank Sum Test, P=0.085, n.s.) and GFP-*Sara*^{F678A} (red; n=24; Mann-Whitney Rank Sum Test, P=0.720, n.s.) with Neur-Gal4, tub-Gal80^{ts}. There is no significant difference in the residence time under these conditions.

All error bars represent standard error of the mean.

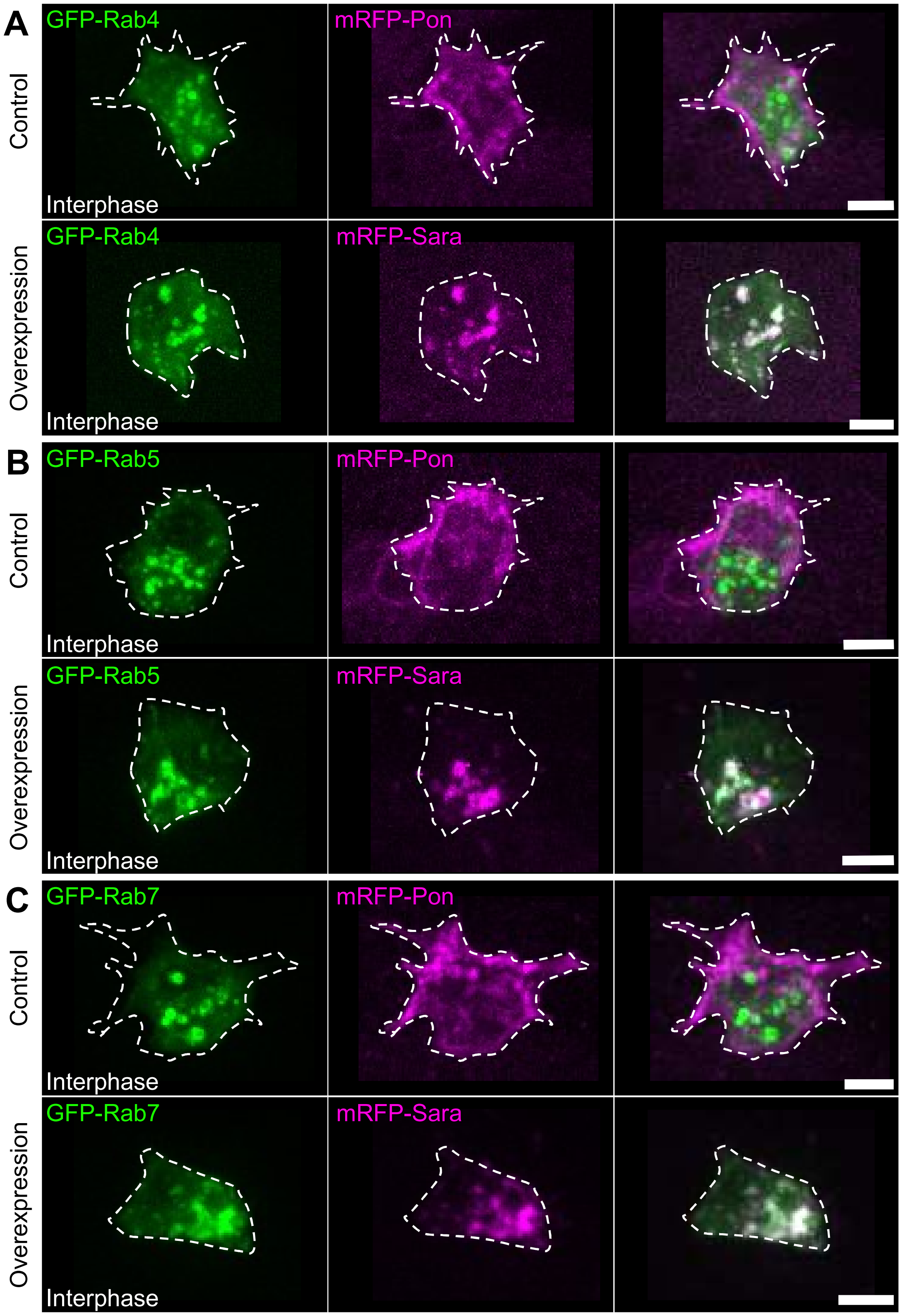

Supplementary Figure 3. Sara endosomes colocalize with Rab4, Rab5 and Rab7 endosomes upon overexpression of Sara in interphase cells.

Supplementary Figure 3. Sara endosomes colocalize with Rab4, Rab5 and Rab7 endosomes upon overexpression of Sara in interphase cells.

(A-C) Images (z projections) of SOPs in interphase expressing GFP-Rab4 (A), GFP-Rab5 (B) or GFP-Rab7 (C) together with mRFP-Pon (upper panel) or mRFP-Sara overexpression (lower panel).

The dashed white lines represent the cell outline; anterior is on the left. Bars: 5 µm.

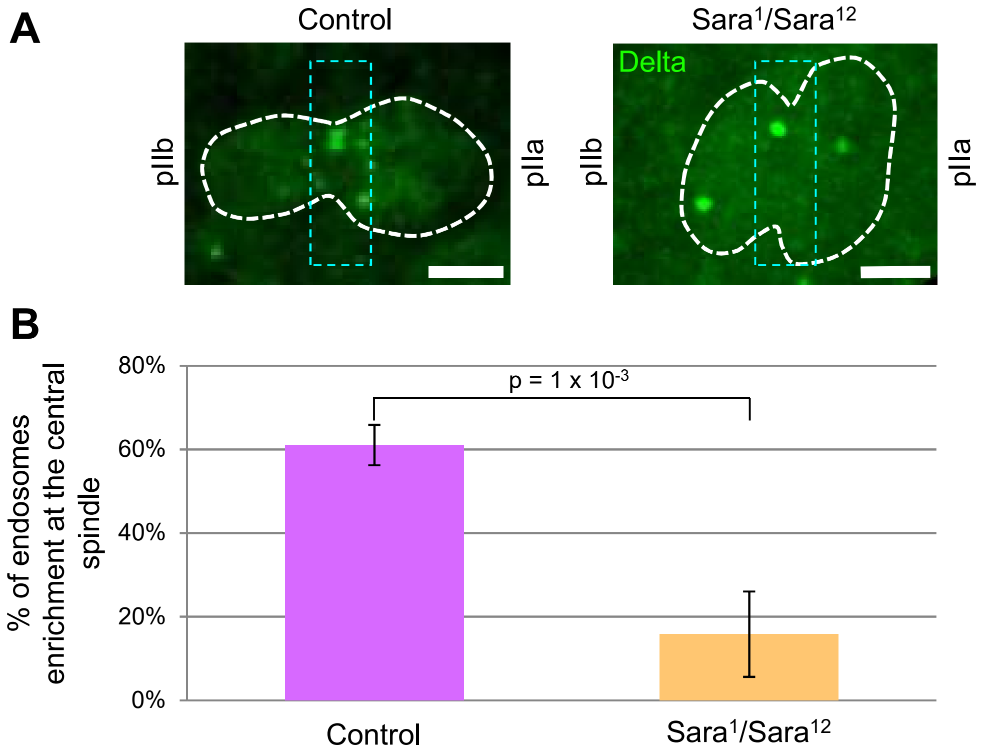

Supplementary Figure 4. Targeting of endogenous Delta positive endosomes to the central spindle is affected in $Sara^1/Sara^{12}$ mutants.

Supplementary Figure 4. Targeting of endogenous Delta positive endosomes to the central spindle is affected in *Sara^{1}/Sara^{12}* mutants.

(A) Immuno-staining (z projections) of SOPs in late cytokinesis stained for Delta in *Sara^{1}/Sara^{12}* mutant animals and in control SOP.

Dashed white lines represent the cell outline and dashed cyan boxes highlight the central spindle; anterior is on the left. Bars: 5 µm.

(B) Percentage of Delta positive endosomes enrichment at the central spindle in control (n=14) and in *Sara^{1}/Sara^{12}* mutant condition (n=10). Mann-Whitney Rank Sum Test, P=0.001. Error bars represent standard error of the mean.

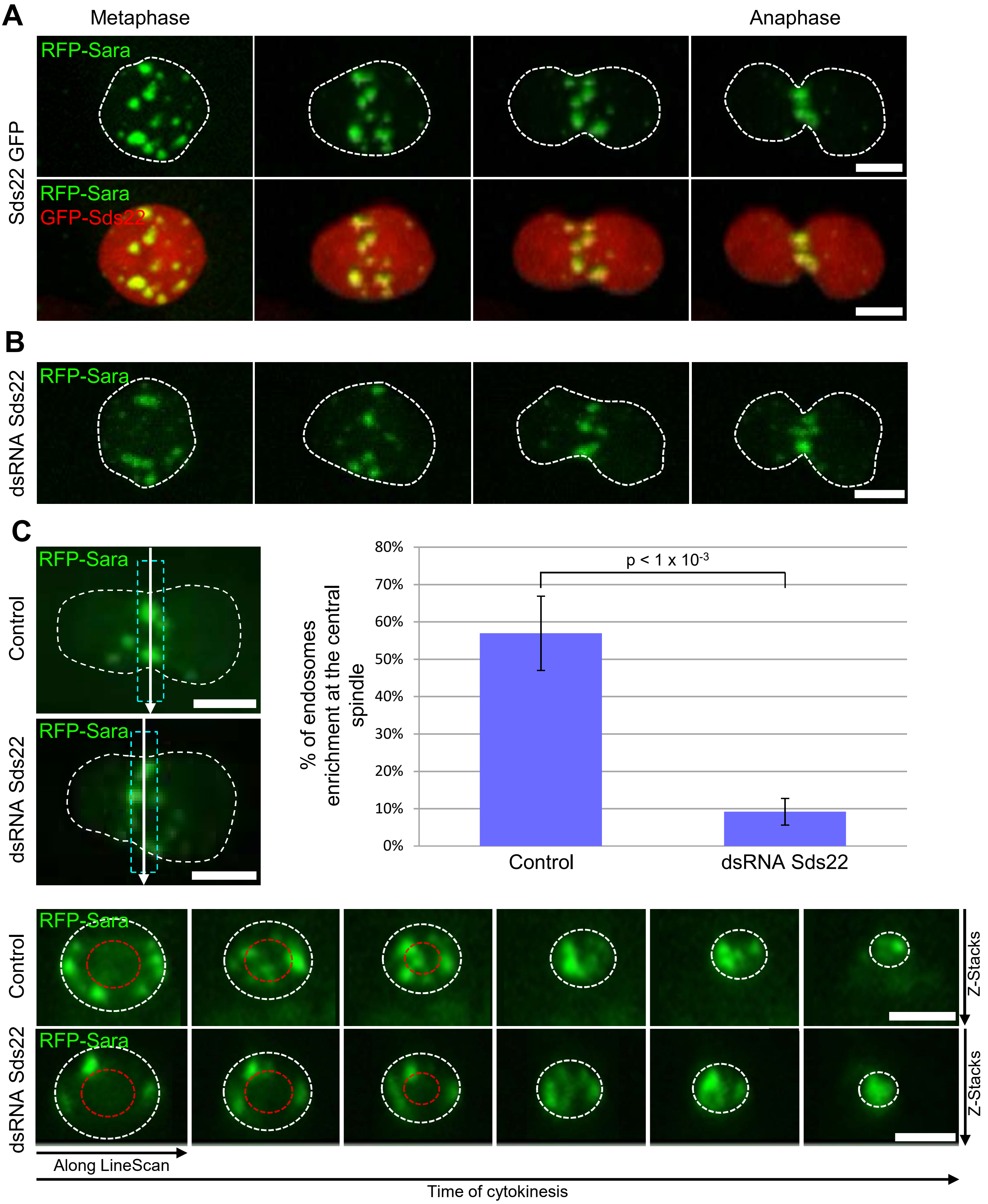

Supplementary Figure 5. Recruitment of Sara endosomes to the central spindle upon depletion and overpression of Sds22.

Supplementary Figure 5. Recruitment of Sara endosomes to the central spindle upon depletion and overpression of Sds22.

(A) Images from time-lapse movies (z projections) of dividing SOP expressing mRFP-Sara and Sds22-GFP in metaphase and anaphase showing the recruitment of Sara endosomes to the central spindle.

(B) Images from time-lapse movies (z projections) of dividing SOP expressing mRFP-Sara and a dsRNA against Sds22 in metaphase and anaphase showing localization of Sara endosomes at the actomyosin ring.

(C) Left upper panel: Image of anaphase SOPs (z projections) from time-lapse movies of a dividing SOP expressing dsRNA-Sds22 with mRFP-Sara or from a control SOP expressing mRFP-Sara. Dashed cyan box, 2 μm-wide region box centered in the central spindle during SOP mitosis where the Z-section is observed in the lower panels.

Right upper panel: Percentage of Sara endosomes enrichment at the central spindle in control (n=12) and in dsRNAi Sds22 conditions (n=6). Mann-Whitney Rank Sum Test, $P<0.001$. Error bars represent standard error of the mean.

Lower panels: Section of the actomyosin ring images of the time-lapse movies shown in the upper panels (Z-sections from the 2 μm-wide dashed cyan boxes). Dashed white circle delimits the furrow outline; dashed red circle delimits the actomyosin ring in the outer annular region and central spindle microtubules in the inner circle. As previously reported, in control cells, Sara endosomes are first targeted to the actin contractile ring at the cleavage plane (monitored by myosin regulatory light chain spaghetti squash localization as shown in Fig. 2E in reference[10]) and subsequently move to the central spindle. In the SOP expressing dsRNA-Sds22, Sara endosomes are localized at the actomyosin ring (outer annular region), but not at the central spindle (inner region). The subsequent spindle targeting event is defective in the mutant.

Dashed white lines represent the cell outlines; anterior is on the left. Bars: 5 μm.

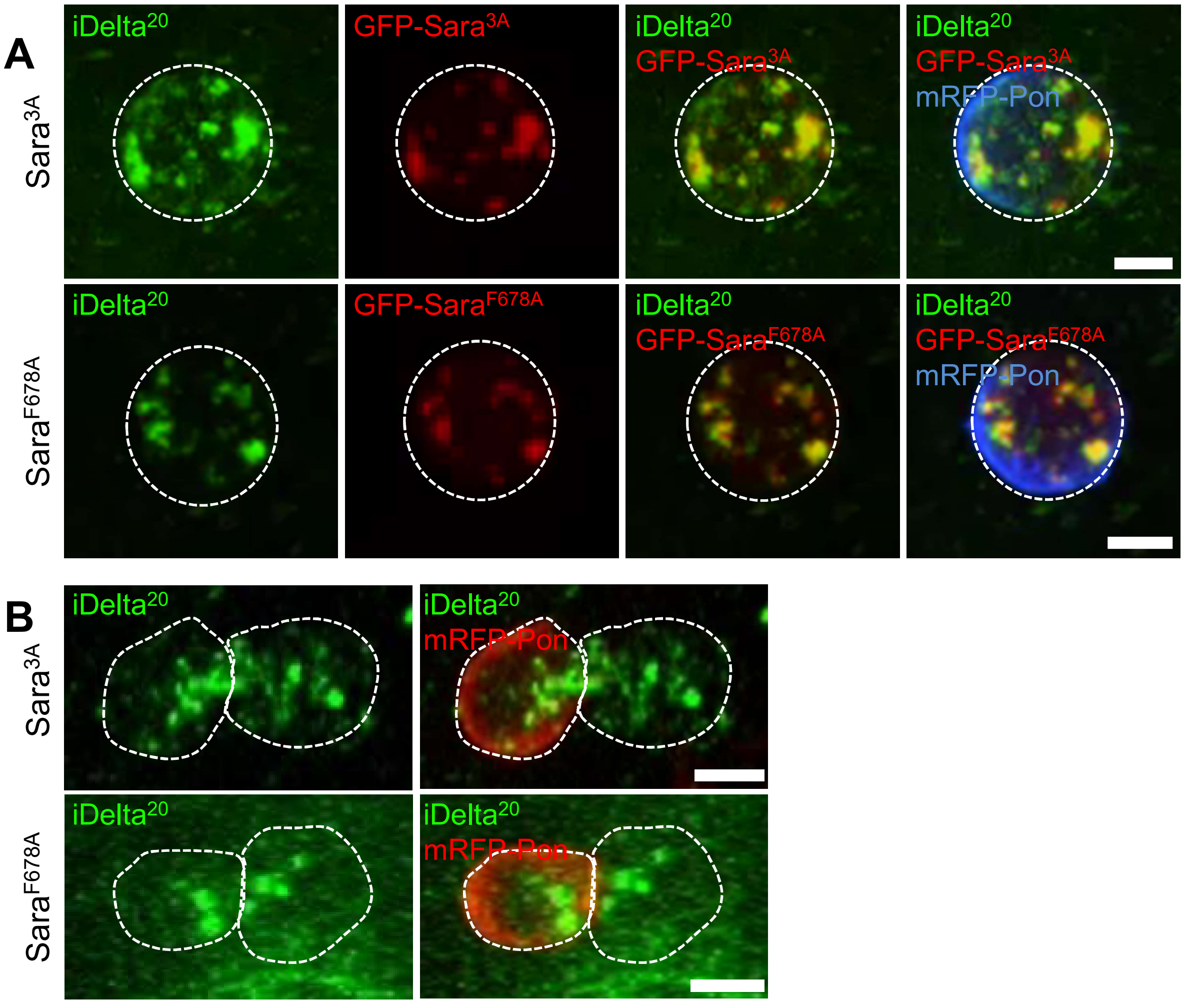

Supplementary Figure 6. iDelta$^{20'}$ endosomes colocalize with Sara endosomes and are symmetrically segregated upon expression of *Sara^{3A}* or *Sara^{F678A}*.

Supplementary Figure 6. iDelta$^{20'}$ endosomes colocalize with Sara endosomes and are symmetrically segregated upon expression of *Sara^{3A}* or *Sara^{F678A}*.

(A) Images from time-lapse movies (z projections) showing Delta uptake (iDelta$^{20'}$) and GFP-*Sara^{3A}* or GFP-*Sara^{F678A}* in metaphase SOPs. Note the colocalization between iDelta$^{20'}$ positive endosomes and Sara endosomes.

(B) Images from time-lapse movies (z projections) showing Delta uptake (iDelta$^{20'}$) in SOPs expressing GFP-*Sara^{3A}* or GFP-*Sara^{F678A}* at abscission. Note symmetrical segregation of iDelta$^{20'}$ positive endosomes in both cases. Dashed white line represents the cell outline; anterior is on the left. Bars: 5 μm.

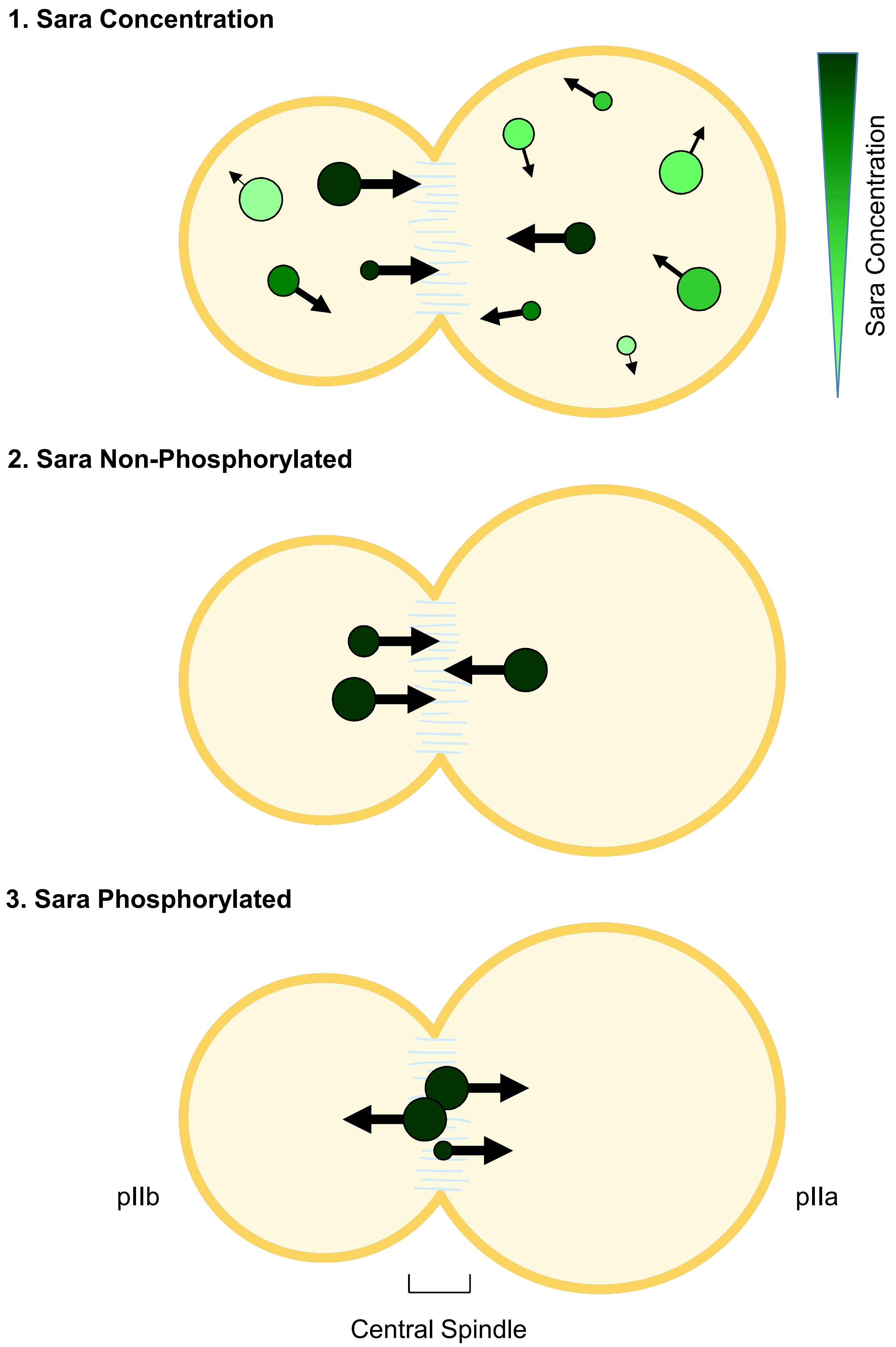

Supplementary Figure 7. Summary of the phenomena discussed in this study: Central spindle targeting of Sara endosomes depends on Sara concentration and phosphorylation.

Supplementary Figure 7. Summary of the phenomena discussed in this study: Central spindle targeting of Sara endosomes depends on Sara concentration and phosphorylation.

1. Sara Concentration: The amount of Sara in endosomes forecasts their targeting to the cleavage plane. Endosomes containing high levels of Sara are displaced more efficiently towards the central spindle compared to endosome with low level.

2. Sara Non-Phosphorylated: The phosphorylation state of Sara determines central spindle targeting. When the three sites are dephosphorylated, endosomes are targeted to the mitotic spindle.

3. Sara Phosphorylated: The phosphorylation state of Sara determines central spindle departure. Phosphorylation of Sara disengages the endosomes from the central spindle allowing the asymmetric departure into the pIIa cell.

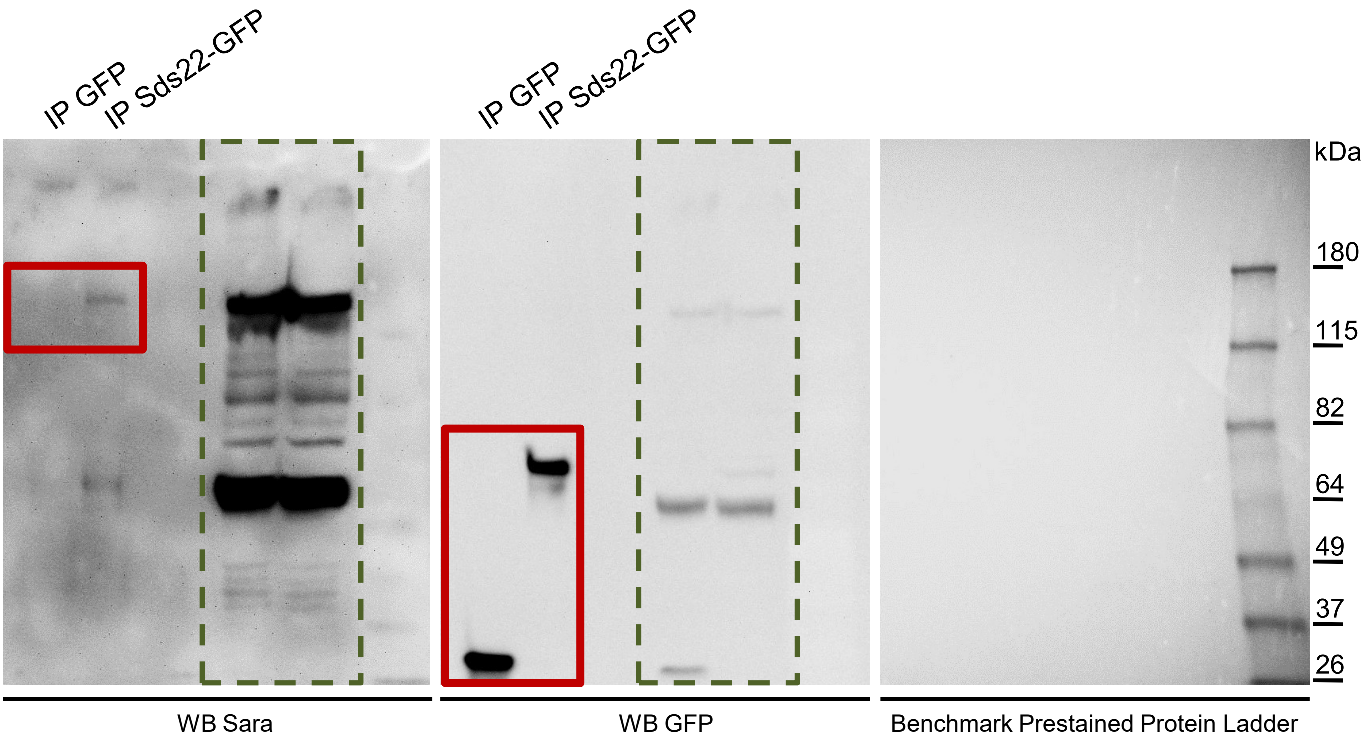

Supplementary Figure 8. Full gel showed in Figure 5.A showing that Sara co-immunoprecipitates with Sds22-GFP, but not with GFP.

Supplementary Figure 8. Full gel showed in Figure. 5A showing that Sara co-immunoprecipitates with Sds22-GFP, but not with GFP.

Red boxes indicate part of the gel showed in Figure. 5A. Dashed green boxes indicate other unrelated experiments which were not showed in this report. Molecular weights indicated in kDa.

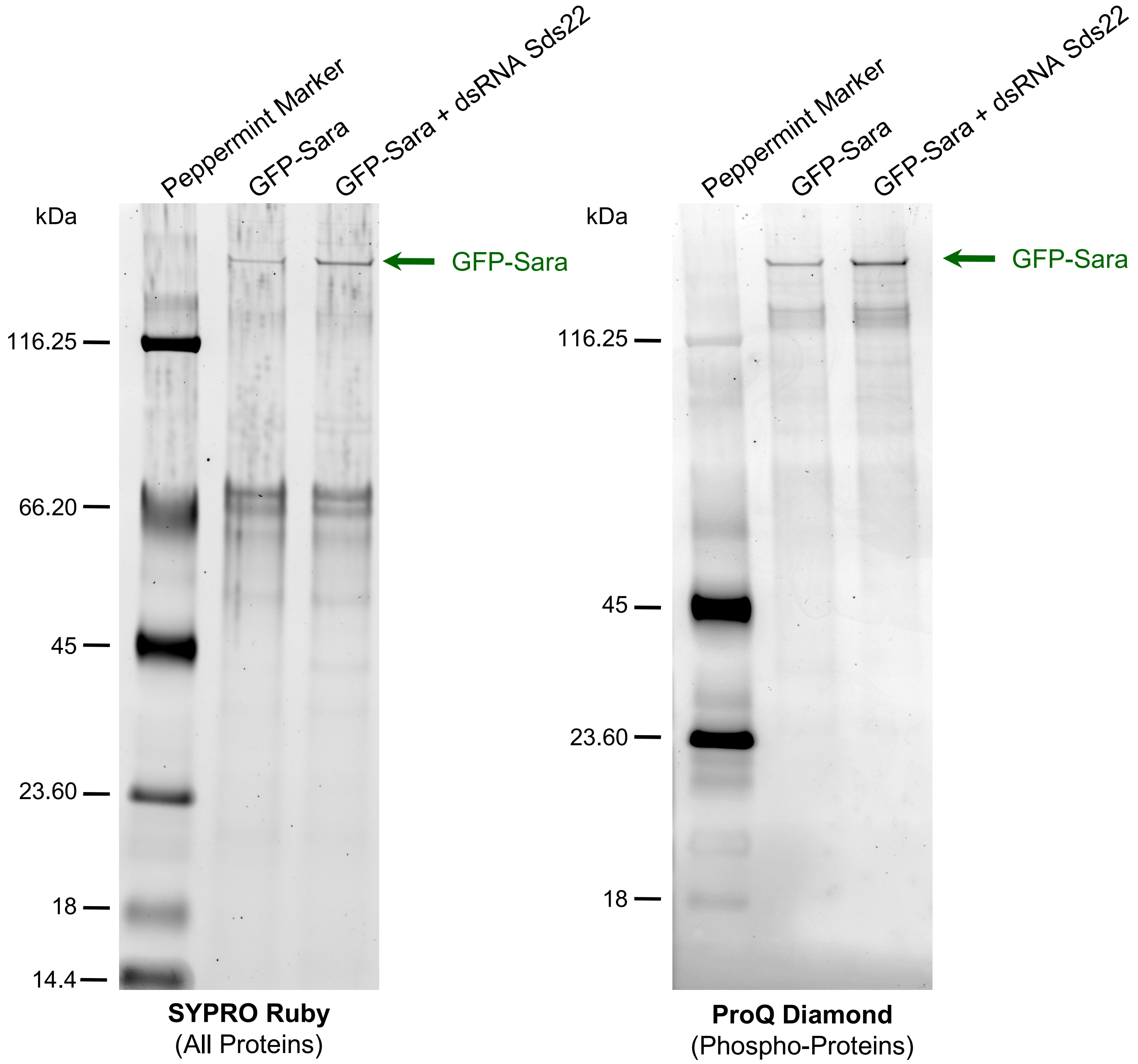

Supplementary Figure 9. Full ProQ Diamond phosphoprotein fluorescent and SYPRO Ruby protein fluorescent gel stains showed in Figure 5.D revealing a 40%-increase in the level of phospho-GFP-Sara upon inhibition of Sds22.

Supplementary Figure 9. Full ProQ Diamond phosphoprotein fluorescent and SYPRO Ruby protein fluorescent gel stains showed in Figure. 5D revealing a 40%-increase in the level of phospho-GFP-Sara upon inhibition of Sds22.

SDS-PAGE followed by stainings of all proteins (SYPRO Ruby; left) and of phospho-proteins (ProQ Diamond; right). Green arrows highlight GFP-Sara bands. Molecular weights indicated in kDa.

Supplementary Methods

Fly lines and fly handling

The UAS-Gal4 system was used for over-expression and RNAi experiments. When using the Gal80^{ts} protein to modulate the levels of expression, animals were kept at 16°C until puparium formation, and then shifted to the temperature specified below for each experiment. Stocks used in this study were: *Neur-Gal4* [27]; *Pnr-Gal4* (Bloomington #3039); *UAS-mRFP-Pon* [7]; *tub-Gal80*^{ts} (Bloomington #7017); *UAS-GFP-Sara* [14]; *Ubi-GFP-Sara* [10]; *Ubi-Pav-GFP* [28]; *Sara*^{1} [14] ; *Sara*^{12} [14]; *Df(2R)48* [29]; *hh-Gal4* [30]; UAS-Sds22-GFP [19]; *UAS-Sds22-RNAi* (VDRC 42051; this RNAi line was validated in [21] and by us by Western blots in flies); UAS-GFP-*Sara*^{3A} (this study); *UAS-GFP-Sara*^{F678A} (this study); *UAS-mRFP-Sara* [10]; UAS-GFP-Rab7; UAS-*GFP-Rab5* [31]; UAS-Neuralized-RNAi (VDRC 108239).

Animals of the following genotypes were studied, and pupae were shifted overnight to the indicated temperatures:

Fig. 1A-F: *w*^{1118}*; tub-Gal80*^{ts} */ +; Neur-Gal4, UAS-GFP-Sara, UAS-mRFP-Pon / +* at 27°C.

Fig. 2A-E: *w*^{1118}*; Neur-Gal4, UAS-mRFP-Pon, Ubi-Pav-GFP / +* at 25°C ("Control"), *w*^{1118}*; Sara*^{12} */ Sara*^{12}*; Neur-Gal4, UAS-mRFP-Pon, Ubi-Pav-GFP /* + at 25°C ("Sara^{12}") and *w*^{1118}*; tub-Gal80*^{ts} */ +; Neur-Gal4, UAS-GFP-Sara, UAS-mRFP-Pon / +* at 27°C (Fig. 2E, "GFP-Sara").

Fig. 3A: *w*^{1118}*; UAS-mRFP-Pon / UAS-GFP-Rab4; Neur-Gal4, tub-Gal80*^{ts} */ +* at 25°C (top panel) and *w*^{1118}*; UAS-GFP-Rab4 / +; Neur-Gal4, tub-Gal80*^{ts} */ UAS-mRFP-Sara* at 25°C (bottom panel). Fig. 3B: *w*^{1118}*; UAS-mRFP-Pon / +; Neur-Gal4, tub-Gal80*^{ts} */ UAS-GFP-Rab5* at 27°C (top panel) and *w*^{1118}*; + / +; Neur-Gal4, tub-Gal80*^{ts}*, UAS-mRFP-Sara / UAS-GFP-Rab5* at 27°C (bottom panel). Fig. 3C: *w*^{1118}*; UAS-mRFP-Pon / UAS-GFP-Rab7; Neur-Gal4, tub-Gal80*^{ts} */ +* at 25°C (top panel) and *w*^{1118}*; UAS-GFP-Rab7 / +; Neur-Gal4, tub-Gal80*^{ts} */ UAS-mRFP-Sara* at 25°C (bottom panel).

Fig. 4: *w*^{1118}*; Df(2R)48 / CyO-GFP; Pnr-Gal4, UAS-DsRed / +* ("Pnr(1)>"; Fig. 4A, Fig. 4D, Fig. 4G and Fig. 4K);

w^{1118}*; Sara*^{1} */ CyO-GFP; Pnr-Gal4, UAS-DsRed/+* ("Pnr(2)>"; Fig. 4K);

w^{1118}; Sara^{12}, UAS-Neur-RNAi / CyO-GFP; Pnr-Gal4, UAS-DsRed / + ("Pnr>Neur^{RNAi} Control (1)"; Fig. 4B, Fig. 4E, Fig. 4H and Fig. 4K); *w^{1118}; Sara^{12}, UAS-Neur-RNAi / CyO-GFP; Pnr-Gal4, UAS-DsRed / +* ("Pnr>Neur^{RNAi} Control (2)"; Fig. 4K); *w^{1118}; Sara^{12}, UAS-Neur-RNAi / Df(2R)48; Pnr-Gal4, UAS-DsRed / +* ("Pnr>Neur^{RNAi}, Sara^{12} / Df(2R)48"; Fig. 4C, Fig. 4F, Fig. 4I and Fig. 4K); *w^{1118}; Sara^{12}, UAS-Neur-RNAi / Sara^{1}; Pnr-Gal4, UAS-DsRed / +* ("Pnr>Neur^{RNAi}, Sara^{1} / Sara^{12}"; Fig. 4J and Fig. 4K); *w^{1118}; Sara^{12}, UAS-Neur-RNAi / Df(2R)48, Ubi-GFP-Sara; Pnr-Gal4, UAS-DsRed / Ubi-GFP-Sara* ("Rescue"; Fig. 4K).

Fig. 5A: *w^{1118}; UAS-Sds22-GFP / +; hh-Gal4, tub-Gal80^{ts} / +* at 29°C (IP Sds22-GFP) and *w^{1118}; + / +; hh-Gal4, tub-Gal80^{ts} / UAS-GFP* at 29°C (IP GFP). Fig. 5B-C: *w^{1118}; UAS-mRFP-Pon / UAS-Sds22-GFP; Neur-Gal4 / +* at 25°C ("Sds22-GFP"), *w^{1118}; UAS-mRFP-Pon / UAS-Sds22-RNAi; Neur-Gal4, tub-Gal80^{ts} / +* at 29°C ("dsRNA Sds22") and *w^{1118}; Neur-Gal4, UAS-mRFP-Pon / +* at 27°C (Fig. 4C, "WT"). Fig. 5D: *w^{1118}; UAS-GFP-Sara / +; hh-Gal4, tub-Gal80^{ts} / +* at 29°C ("GFP-Sara") and *w^{1118}; UAS-GFP-Sara / UAS-Sds22-RNAi; hh-Gal4, tub-Gal80^{ts} / +* at 29°C ("GFP-Sara + dsRNA Sds22").

Fig. 6A-E: *w^{1118}; tub-Gal80^{ts} / +; Neur-Gal4, UAS-GFP-Sara, UAS-mRFP-Pon / +* at 27°C ("GFP-Sara"), *w^{1118}; UAS-mRFP-Pon / +; Neur-Gal4, tub-Gal80^{ts} / UAS-GFP-Sara^{3A}* at 27°C ("GFP-Sara^{3A}") and *w^{1118}; UAS-mRFP-Pon / UAS-GFP-Sara^{F678A}; Neur-Gal4, tub-Gal80^{ts} / +* at 29°C ("GFP-Sara^{F678A}"). Fig. 6F-G: *w^{1118}* at 25°C then 29°C (shift of temperature at 3rd instar larval stage), *w^{1118};; Neur-Gal4, tub-Gal80^{ts} / UAS-GFP-Sara* at 25°C then 29°C (shift of temperature at 3rd instar larval stage; "GFP-Sara"), *w^{1118};; Neur-Gal4, tub-Gal80^{ts} / UAS-GFP-Sara^{3A}* at 25°C then 29°C (shift of temperature at 3rd instar larval stage; "GFP-Sara^{3a}") and *w^{1118}; UAS-GFP-Sara^{F678A} / +; Neur-Gal4, tub-Gal80^{ts} / +* at 25°C then 29°C (shift of temperature at 3rd instar larval stage; "GFP-Sara^{F678A}").

Supplementary Fig. 2A: *w; UAS GFP-Sara / +; UAS mRFP-Pon, Neur-Gal4, tub-Gal80^{ts} / +* at 25°C then 29°C (shift of temperature at the 3rd instar larval stage) ("GFP-Sara"), *w; UAS GFP-Sara^{3A} / +; UAS mRFP-Pon, Neur-Gal4, tub-Gal80^{ts} / +* at 25°C then 29°C (shift of temperature at the 3rd instar larval stage) ("GFP-Sara^{3A}") and *w; UAS GFP-Sara^{F678A} / +; UAS mRFP-Pon, Neur-Gal4, tub-Gal80^{ts} / +* at 25°C then 29°C (shift of temperature at the 3rd instar

larval stage) ("GFP-Sara^{F678A}"). Supplementary Fig 2B-E: *w; UAS GFP-Sara / +; UAS mRFP-Pon, Neur-Gal4, tub-Gal80*^{ts} / + at 25°C ("GFP-Sara"), *w; UAS GFP-Sara*^{3A} */ +; UAS mRFP-Pon, Neur-Gal4, tub-Gal80*^{ts} / + at 25°C ("GFP-Sara^{3A}") and *w; UAS GFP-Sara*^{F678A} */ +; UAS mRFP-Pon, Neur-Gal4, tub-Gal80*^{ts} / + at 25°C ("GFP-Sara^{F678A}").

Supplementary Fig. 3A: *w*^{1118}*; UAS-mRFP-Pon / UAS-GFP-Rab4; Neur-Gal4, tub-Gal80*^{ts} / + at 25°C (top panel) and *w*^{1118}*; UAS-GFP-Rab4 / +; Neur-Gal4, tub-Gal80*^{ts} */ UAS-mRFP-Sara* at 25°C (bottom panel). Supplementary Fig. 3B: *w*^{1118}*; UAS-mRFP-Pon / +; Neur-Gal4, tub-Gal80*^{ts} */ UAS-GFP-Rab5* at 25°C (top panel) and *w*^{1118}*; + / +; Neur-Gal4, tub-Gal80*^{ts}*, UAS-mRFP-Sara / UAS-GFP-Rab5* at 25°C (bottom panel). Supplementary Fig. 3C: *w*^{1118}*; UAS-mRFP-Pon / UAS-GFP-Rab7; Neur-Gal4, tub-Gal80*^{ts} / + at 25°C (top panel) and *w*^{1118}*; UAS-GFP-Rab7 / +; Neur-Gal4, tub-Gal80*^{ts} */ UAS-mRFP-Sara* at 25°C (bottom panel).

Supplementary Fig. 4A-B: *w*^{1118}*; Sara*^{1} */ Sara*^{12} at 25°C ("Sara^{1}/Sara^{12}") and *w*^{1118}*; UAS Pavarotti GFP; UAS-mRFP-Pon, Neur-Gal4 / TM6B* at 25°C ("Control").

Supplementary Fig. 5A: *w*^{1118}*; UAS-mRFP-Sara / UAS-Sds22-GFP; Neur-Gal4, tubGal80*^{ts} / + at 25°C ("Sds22 GFP"). Supplementary Fig. 5B: *w*^{1118}*; UAS-mRFP-Sara / UAS-Sds22-RNAi; Neur-Gal4, tub-Gal80*^{ts} / + at 29°C ("dsRNA Sds22"). Supplementary Fig. 5C *w*^{1118}*; UAS-mRFP-Sara / +; Neur-Gal4, tub- Gal80*^{ts} */ UAS GFP* at 27°C ("Control") and *w*^{1118}*; UAS-mRFP-Sara / UAS-Sds22-RNAi; Neur-Gal4, tub-Gal80*^{ts} / + at 29°C ("dsRNA Sds22").

Supplementary Fig. 6A-B: *w; UAS GFP-Sara*^{3A} */ +; UAS mRFP-Pon, Neur-Gal4, tub-Gal80*^{ts} / + at 27°C ("GFP-*Sara*^{3A}") and *w; UAS GFP-Sara*^{F678A} */ +; UAS mRFP-Pon, Neur-Gal4, tub-Gal80*^{ts} / + at 29°C ("GFP-*Sara*^{F678A}").

Scanning Electron Microscopy (SEM)

Quantification of the Neur phenotypes:

To study the relevance of Sara in Notch signaling during SOP cell-fate assignation, we followed the rationale previously established[15]. We used a partial depletion of Neuralized in the center of the notum (using Pnr>*Neur*^{RNAi}), which allows many sensory organs to still undergo asymmetric cell fate

assignation and to develop, as in *wild-type*, into structures containing at least the two external cells. However, these remaining structures are absent in *Pnr>Neur^{RNAi}, Sara^{12} / Df(2R)48* transheterozygotes mutants, leading to a naked cuticle (Fig. 4C,F,I and Fig. 4K). Importantly, in these transheterozygotes mutants, the number of SOP is 5 times that of control ("Pnr>") animals (Fig. 4C, and see below), ruling out a potential early SOP specification defect that could have led to a loss of SOP mother cells. These results were confirmed using another Sara mutant background (*Sara^{1} / Sara^{12}*). These effects derive from the absence of Sara function, since *Sara^{1}* is a point mutation in the FYVE domain of Sara. Both *Sara^{12}* and Df(2R)48 do however affect the expression of a neighbouring gene, syndecan:

Quantification of the cell-fate phenotypes within the SOP lineage in *Neur^{RNAi}* flies (Fig. 4K) was performed as previously described[15] on scanning electron microscopy images.

To ensure that the absence of bristles in *Pnr>Neur^{RNAi}, Sara^{12} / Df(2R)48* transheterozygote mutant flies was not due to an early SOP specification defect (i.e. the fly central notum could have been bald because SOPs were never specified there), we performed immunostainings with the neurogenesis marker Hindsight at the one-cell stage of the SOP (Fig. 4A-C). Quantifications reveal an increase of SOP numbers in control *Sara^{12}, UAS-Neur-RNAi / CyO-GFP*; *Pnr-Gal4, UAS-dsRed /* + flies compared to "Pnr>" control flies due to the knock-down of Neuralized (498±52 versus 136±8, N=4 flies per condition, ANOVA test, P<0.001), which is further increased in *Pnr>Neur^{RNAi}, Sara^{12} / Df(2R)48* transheterozygote mutants (668+/-38 versus 136±8, N=4 flies per condition, ANOVA test, P=0.027). Furthermore, immunostaining with the neuron-specific marker Elav revealed clusters of Elav-positive neurons (Fig. 4E-F).

Clonal analysis has been previously used to address whether Neuralized acts in pIIa or pIIb exclusively[6]. Upon initiation of a *neuralized* mutant clone in the SOP, only one of the two daughter cells is homozygous mutant, either pIIa or pIIb[6]. Under these conditions, no phenotype was observed whether pIIa or pIIb is mutant[6]. When pIIb is mutant, it can still inherit from the SOP the Neuralized anterior cortical localization domain (crescent) and this could

rescue the loss of the *neuralized* functional gene; because there is also no mutant phenotype when pIIa is mutant, this might indicate that Neuralized act in pIIb, where the crescent is seen[6]. However, Neuralized is a cytosolic factor that is enriched in the anterior cortex, while the largest pool of the Neuralized protein can remain cytosolic and present in the mutant pIIa and pIIb ("perdurance"). Clonal analysis is therefore too slow to address the relevance of the biased localization of Neuralized at the pIIb cortex or Sara endosomes to the pIIa during asymmetric Notch signaling: it only informs about the perdurance of the protein. The same reasoning applies to Sara, which is a cytosolic protein that is enriched on endosomes.

Quantification of the $Sara^{3A}$ and $Sara^{F678A}$ phenotypes:

To study the relevance of Sara phosphorylation in Notch signaling during SOP cell-fate assignation, we studied the GFP-$Sara^{3A}$ and GFP-$Sara^{F678A}$ expressing animals (See DNA constructs). The phenotype can be directly seen by itself in the Sara mutant conditions in the postorbital bristles without sensitizing the system for Notch signaling with $Neuralized^{RNAi}$ as the postorbital bristles have been previously shown to be particularly sensitive in mutants for asymmetric division[8]. We expressed UAS-GFP-Sara, UAS-GFP-$Sara^{3A}$ and UAS-GFP-$Sara^{F678A}$ under the Neur-Gal4 driver with temporal control by the $Gal80^{ts}$ system[19]. After hatching, embryos were kept at 25°C, then shifted at the 3rd instar larval stage to 29°C until pupariation (for GFP-Sara and GFP-$Sara^{3A}$) or adult flies (for GFP-$Sara^{F678A}$). Indeed, in *w^{1118};; Neur-Gal4, tub-$Gal80^{ts}$ / UAS-GFP-Sara* and in *w^{1118};; Neur-Gal4, tub-$Gal80^{ts}$ / UAS-GFP-$Sara^{3A}$* expressing animals, we observed late pupal death. We dissected the pharate adults to study the post-orbital. In the case of *w^{1118}; UAS-GFP-$Sara^{F678A}$ / +; Neur-Gal4, tub-$Gal80^{ts}$* / + viable adult flies were studied.

Quantification of the cell-fate phenotypes within the post-orbital lineage in GFP-$Sara^{3A}$ and GFP-$Sara^{F678A}$ genotypes (Fig. 6F-G) was performed on scanning electron microscopy images. In both $Sara^{3A}$ and $Sara^{F678A}$ mutants, supernumerary sockets were observed in the lineages: 88% of double sockets in the lineages for $Sara^{3A}$ and 82% of double sockets in the lineages for $Sara^{F678A}$. We have found a milder version of this phenotype by

overexpressing *wild-type* Sara: 34% of double sockets in the lineages and none double sockets in white control flies (Fig. 6F-G).

Fluorescence Recovery After Photobleaching (FRAP)

FRAP experiments with GFP-Sara, GFP-*Sara*^{3A} and GFP-*Sara*^{F678A} (Supplementary Fig. 2B-E) were performed on a 3i Marianas spinning disk setup described above (63× NA 1.4 oil objective) equipped with a Micropoint Photomanipulation hardware driven by Slidebook 6.0. A region of interest (ROI) was drawn onto a single endosome, bleached and recovery was monitored by spinning disk confocal imaging. Owing to the fast recovery of GFP-Sara (timescale of few seconds), recovery was monitored in 2D (one z plane) to maximize the frame-rate.

FRAP movies were processed as follows: GFP-Sara and GFP-Sara mutant signals within the bleached ROI was integrated overtime. Raw, unnormalized fluorescence intensity was then fitted to the equation:

$$I(t) = B + A\left(1 - e^{\frac{-(t-t_0)}{\tau}}\right)$$

In this equation, 1/τ is an estimate of the k_{off} of GFP–Sara on endosomes.

Intensity was then normalized using the formula:

$$I_{\text{norm}}(t) = \frac{I(t) - B}{A}$$

With I(t), the experimental integrated intensity at time point t; A and B are fit parameters from the fit equation above. This $I_{\text{norm}}(t)$ is what is plotted in Supplementary Fig. 2B-D. τ values were then averaged over several recovery curves for each mutant condition. Average τ as in Supplementary Fig. 2E: $\tau_{\text{GFP-Sara}^{WT}}$ = 2.95 ± 0.54 s, n=10, $\tau_{\text{GFP-Sara}^{3A}}$ = 3.94 ± 0.34 s, n=19 and $\tau_{\text{GFP-Sara}^{F678A}}$ = 2.51 ± 0.20 s, n=24.

Image processing

Image processing was performed using Fiji[32] and Matlab (MathWorks).

Detection and tracking of endosomes

Endosomes were detected using two different algorithms depending on the size and shape of the endosome. See also Supplementary Fig. 1.

i) Initially all endosomes were detected by fitting 2D Gaussian functions to the pixel intensity profiles of the images as previously described[26]. From the Gaussian fit, several properties of the endosome were obtained, e.g. the position of the fluorescence centroid and the (apparent) radius (Full Width Half Maximum of the Gaussian).
ii) When an endosome radius was found to be larger than the diffraction limit, the result of the Gaussian approach was discarded for this specific endosome. Instead, an intensity threshold was applied to segment the endosome and the endosome position was then determined by calculating the position of the center of mass.

Endosome tracking was then performed using a modified Vogel-algorithm[26]. This algorithm requires an estimation of the motility of the endosomes (the 'diffusion' constant). Because endosome velocities varied significantly during the movie, this caused trajectories to be terminated prematurely when the endosome displacement was too long. These sub-trajectories were manually linked afterwards.

GFP-Sara levels in endosomes

GFP-Sara levels were calculated by dividing the fluorescent intensity in an endosome by the surface area of that endosome. For this purpose, endosomes were considered to be spherical with a diameter equal to the FWHM (Full Width Half Maximum) obtained from the 2D Gaussian fit, or from an approximation to the real shape when no 2D Gaussian fit was available. If an endosome was detected in multiple planes, fluorescence intensities of the endosome in these planes was summed. To segment the population of endosomes according to high and low Sara levels, a threshold (444 au) was chosen by classifying the Sara endosome collection into two groups of approximately the same number of endosomes.

Detection of the Pon-crescent and the division plane

Before cytokinesis, the Pon-crescent was used to determine the division plane that will separate the two daughter cells. We developed an algorithm to automatically detect the Pon-crescent in each image plane and calculate the division plane using the edges of the crescent, see Supplementary Fig. 1.

Cumulative displacement

For each time point in each endosomal trajectory, the distance r of the endosome to the putative division plane was calculated. Considering this distance to the division plane, the displacement d towards the division plane for a time point t was computed by comparing the distance r_t at time point t to the distance of the endosome 10 time points later: $d=r_{t+10}-r_t$. Thus, only endosomal trajectories with a duration of more than 10 time points were considered for the calculation of the cumulative displacement. This filters out fluctuations on short time scales, while relatively short trajectories (e.g. from endosomes with low Sara levels) are still taken into account. Displacements towards the division plane are positive; displacements away from the division plane, negative. For each endosome track, the cumulative displacement at a time point t is the sum of consecutive displacements from the beginning to that time point (for two example tracks, see Fig. 1E). Then, the average displacement of all endosomes is calculated for each time point. From this the average cumulative displacement of all the trajectories is calculated and shown in Figure 1F.

Endosomal asymmetry

The quantification of the proportion of endosomes targeted to the pIIa and the pIIb daughter cells (Fig. 2D, Fig. 4C and Fig. 5E) was performed as described in[1]. Briefly, the total endosomal intensity in the pIIa and the pIIb cells (I_{pIIa} and I_{pIIb}, respectively) was measured by integrating total intensity values in each slice of the first z-stack after abscission, after having subtracted the background and thresholded the endosomes; the percentage of endosomes in the pIIa cell was finally computed as $I_{pIIa} / (I_{pIIa} + I_{pIIb})$.

Enrichment at the central spindle

Enrichments at the central spindle (Fig. 1C-D, Fig. 2C, Fig. 2E and Fig. 5C-D) were calculated as in[11]. Cells were registered in time by setting the first time point after abscission as the origin (t=0). The central spindle region was

defined at each time point as the volume of the SOP included in a 2 µm-wide box centered on the cleavage furrow; this box approximately encompassed the whole length of the central spindle. The mean relative volume of the central spindle region at each time point was measured using SOPs expressing cytosolic GFP: at each time point, the volume of the SOP included in the central spindle region was measured, and divided by the total volume of the SOP at that time point; results were finally averaged on 4-13 cells. Then, for each cell for which the endosome enrichment at the central spindle was analyzed, first, the fluorescence background level was subtracted and vesicles were manually thresholded. At each time point, the intensity of endosomes in the central spindle region was integrated over the z stack, and the value obtained was divided by the integrated intensity of endosomes outside of this region, giving the in/out ratio. The enrichment on the central spindle was finally calculated by normalizing the in/out ratio by the mean relative volume of the central spindle region at the corresponding time point. Finally, enrichments calculated at each time point were averaged between cells, and plotted over time; spline interpolation was used to resample the cells that had a sampling frequency different than 20s.

The decay of the time of departure (Fig. 1D and 5D) was measured the following way. First, the fold enrichment at the central spindle was measured as explained above. Then, the departure phase was defined: in the time period between -260s and +100s (relative to abscission), the departure phase start was defined as the time point in which the fold enrichment reached its maximal value; the departure phase end was defined as 100s after abscission. During the departure phase, the fold enrichment was fitted to an exponential, according to the equation $\text{Enrichment}(t)=a.\exp(-t/\tau)+b$; with t, time and τ, the decay time. These decay times τ were finally averaged between cells (n=17 cells in 3 animals for GFP-Sara and n=15 cells in 4 animals for GFP-Sara^{3A}), and presented as mean ± standard error of the mean.

Enrichments at the central spindle in Supplementary Fig. 4B were calculated using custom codes written for Fiji/ImageJ. The central spindle region was defined as the volume of the SOP included in a 2 µm-wide ROI (Region Of Interest) centered on the cleavage furrow; this ROI approximately

encompassed the whole length of the central spindle and was included within the cell contour (made by Hindsight or Pon channel). For each cell, the fluorescence was manually thresholded and the intensity of endosomes in the central spindle region was integrated over the z stacks. The percentage of endosomes enrichment at the central spindle was then computed as the enrichment of the density of the delta signal in the central spindle region relative to the entire SOP cell according to:

$$\text{\% of endosomes enrichment at the central spindle} = \frac{\text{IntDen}_{\text{Inside Central Spindle Region}}}{\text{IntDen}_{\text{Inside Central Spindle Region}} + \text{IntDen}_{\text{Outside Central Spindle Region}}} \times 100$$

where IntDen is the Integrated Density function of ImageJ/Fiji[32] (product of Area and Mean Gray Value). Finally, the calculated enrichments were averaged between cells (n=14 cells for the Control and n=10 cells for *Sara*^{1}/*Sara*^{12}) and presented as mean ± standard error of the mean.

Enrichment at the central spindle vs actin ring from Z-Sections

Enrichments at the central spindle in Supplementary Fig. 5C were calculated using custom codes written for Fiji/ImageJ. Z-sections movies were extracted from a 2 µm-wide boxes centered at the central spindle of time-lapse movies in order to observe the actomyosin ring contraction. In the Z-section images, the furrow outline was defined as a circle ROI (Region Of Interest) centered on the cleavage furrow, the central spindle microtubules region was defined as an inner circle ROI with the same center as the ring outline circle and the actomyosin ring was defined as an annular ROI confined in between the two previous circle ROIs. For each cell, the fluorescence was manually thresholded and the intensity of endosomes in the central spindle inner circle region and in the actin ring annular region was measured when the furrow radius was between 3µm < furrow radius < 6µm. The percentage of endosomes enrichment at the central spindle was then computed as the enrichment of the Sara signal in the central spindle region relative to the entire SOP cell according to:

$$\text{\% of endosomes enrichment at the central spindle} = \frac{\text{IntDen}_{\text{Central Spindle Region}}}{\text{IntDen}_{\text{Central Spindle Region}} + \text{IntDen}_{\text{Actin Ring Region}}} \times 100$$

where IntDen is the Integrated Density function of ImageJ/Fiji[32] (product of Area and Mean Gray Value). Finally, the calculated enrichments were averaged between cells (n=12 cells for the Control and n=6 cells for dsRNA Sds22) and presented as mean ± standard error of the mean.

Expression levels in GFP-Sara, GFP-Sara3A and GFP-SaraF678A

Identical acquisition setup for live imaging was performed for each condition (GFP-Sara, GFP-*Sara*^{3A} and GFP-*Sara*^{F678A}): Consecutive z stacks were acquired with 10ms exposure time for the GFP channel and 100ms exposure time for the RFP channel. Maximum intensity z projections of SOPs in metaphase have been considered. GFP-Sara levels in each condition were calculated by dividing the total fluorescent intensity of a SOP by the surface area of that SOP. The fluorescent intensity was then normalized by the mRFP-Pon signal.

Statistical analysis

Statistical analyses were performed using R (http://CRAN.R-project.org) (Fig. 2D, 5C and 6E) or the SigmaStat 3.5 software (Systat; Fig. 4K, S2A, S2E S4B and S5C). P-values indicated on Fig. 2D, 4C and 5E correspond to Student's t tests (applying the Bonferroni correction in the case of Fig. 5C and 5E), which have been performed after the normality of the distributions studied was verified using Kolmogorov-Smirnov tests. P-values indicated in Fig. 4K correspond to Dunn post-hoc tests performed after a non-parametric Kruskal-Wallis test, the variables being not normal. P-values indicated in Supplementary Fig. 2A correspond to a One Way Analysis of Variance test. P-values indicated in Supplementary Fig. 2E correspond to a Mann-Whitney Rank Sum Test run after a non-parametric Kruskal-Wallis test, the variables being not normal. P-values indicated in Supplementary Fig. 4B and S5C correspond to a Mann-Whitney Rank Sum Test.

All representations on graphs and values in the text are given as mean ± standard error of the mean.

Supplementary References

1 Loubery, S. & Gonzalez-Gaitan, M. Monitoring notch/delta endosomal trafficking and signaling in Drosophila. *Methods Enzymol* **534**, 301-321, doi:10.1016/B978-0-12-397926-1.00017-2 (2014).

2 Gho, M., Lecourtois, M., Geraud, G., Posakony, J. W. & Schweisguth, F. Subcellular localization of Suppressor of Hairless in Drosophila sense organ cells during Notch signalling. *Development* **122**, 1673-1682 (1996).

3 Gho, M., Bellaiche, Y. & Schweisguth, F. Revisiting the Drosophila microchaete lineage: a novel intrinsically asymmetric cell division generates a glial cell. *Development* **126**, 3573-3584 (1999).

4 Knoblich, J. A. Asymmetric cell division: recent developments and their implications for tumour biology. *Nat Rev Mol Cell Biol* **11**, 849-860, doi:10.1038/nrm3010 (2010).

5 Furthauer, M. & Gonzalez-Gaitan, M. Endocytic regulation of notch signalling during development. *Traffic* **10**, 792-802, doi:10.1111/j.1600-0854.2009.00914.x (2009).

6 Le Borgne, R. & Schweisguth, F. Unequal segregation of Neuralized biases Notch activation during asymmetric cell division. *Dev Cell* **5**, 139-148 (2003).

7 Emery, G. *et al.* Asymmetric Rab 11 endosomes regulate delta recycling and specify cell fate in the Drosophila nervous system. *Cell* **122**, 763-773, doi:10.1016/j.cell.2005.08.017 (2005).

8 Berdnik, D., Torok, T., Gonzalez-Gaitan, M. & Knoblich, J. A. The endocytic protein alpha-Adaptin is required for numb-mediated asymmetric cell division in Drosophila. *Dev Cell* **3**, 221-231 (2002).

9 Hutterer, A. & Knoblich, J. A. Numb and alpha-Adaptin regulate Sanpodo endocytosis to specify cell fate in Drosophila external sensory organs. *EMBO Rep* **6**, 836-842, doi:10.1038/sj.embor.7400500 (2005).

10 Coumailleau, F., Furthauer, M., Knoblich, J. A. & Gonzalez-Gaitan, M. Directional Delta and Notch trafficking in Sara endosomes during asymmetric cell division. *Nature* **458**, 1051-1055, doi:10.1038/nature07854 (2009).

11 Loubery, S. *et al.* Uninflatable and Notch control the targeting of Sara endosomes during asymmetric division. *Curr Biol* **24**, 2142-2148, doi:10.1016/j.cub.2014.07.054 (2014).

12 Montagne, C. & Gonzalez-Gaitan, M. Sara endosomes and the asymmetric division of intestinal stem cells. *Development* **141**, 2014-2023, doi:10.1242/dev.104240 (2014).

13 Kressmann, S., Campos, C., Castanon, I., Furthauer, M. & Gonzalez-Gaitan, M. Directional Notch trafficking in Sara endosomes during asymmetric cell division in the spinal cord. *Nat Cell Biol* **17**, 333-339, doi:10.1038/ncb3119 (2015).

14 Bokel, C., Schwabedissen, A., Entchev, E., Renaud, O. & Gonzalez-Gaitan, M. Sara endosomes and the maintenance of Dpp signaling levels across mitosis. *Science* **314**, 1135-1139, doi:10.1126/science.1132524 (2006).

15 Derivery, E. *et al.* Polarized endosome dynamics by spindle asymmetry during asymmetric cell division. *Nature* **528**, 280-285, doi:10.1038/nature16443 (2015).
16 Hamel, S., Fantini, J. & Schweisguth, F. Notch ligand activity is modulated by glycosphingolipid membrane composition in Drosophila melanogaster. *J Cell Biol* **188**, 581-594, doi:10.1083/jcb.200907116 (2010).
17 Tsukazaki, T., Chiang, T. A., Davison, A. F., Attisano, L. & Wrana, J. L. SARA, a FYVE domain protein that recruits Smad2 to the TGFbeta receptor. *Cell* **95**, 779-791 (1998).
18 Bennett, D. & Alphey, L. PP1 binds Sara and negatively regulates Dpp signaling in Drosophila melanogaster. *Nat Genet* **31**, 419-423, doi:10.1038/ng938 (2002).
19 Grusche, F. A. *et al.* Sds22, a PP1 phosphatase regulatory subunit, regulates epithelial cell polarity and shape . *BMC developmental biology* **9**, 14, doi:10.1186/1471-213X-9-14 (2009).
20 Kunda, P. *et al.* PP1-mediaed moesin dephosphorylation couples polar relaxation to mitotic exit. *Curr Biol* **22**, 231-236, doi:10.1016/j.cub.2011.12.016 (2012).
21 Rodrigues, N. T. *et al.* Kinetochore-localized PP1-Sds22 couples chromosome segregation to polar relaxation. *Nature* **524**, 489-492, doi:10.1038/nature14496 (2015).
22 Posch, M. *et al.* Sds22 regulates aurora B activity and microtubule-kinetochore interactions at mitosis. *The Journal of cell biology* **191**, 61-74, doi:10.1083/jcb.200912046 (2010).
23 Wurzenberger, C. *et al.* Sds22 and Repo-Man stabilize chromosome segregation by counteracting Aurora B on anaphase kinetochores. *The Journal of cell biology* **198**, 173-183, doi:10.1083/jcb.201112112 (2012).
24 Ceulemans, H. & Bollen, M. Functional diversity of protein phosphatase-1, a cellular economizer and reset button. *Physiological reviews* **84**, 1-39, doi:10.1152/physrev.00013.2003 (2004).
25 Bodenmiller, B. *et al.* PhosphoPep--a phosphoproteome resource for systems biology research in Drosophila Kc167 cells. *Molecular systems biology* **3**, 139, doi:10.1038/msb4100182 (2007).
26 Holtzer, L. & Schmidt, T. The tracking of individual molecules in cells and tissues. *Single Particle Tracking and Single Molecule Energy Transfer*, Wiley-VCH, Weinheim, Germany. (2010).
27 Bellaiche, Y., Gho, M., Kaltschmidt, J. A., Brand, A. H. & Schweisguth, F. Frizzled regulates localization of cell-fate determinants and mitotic spindle rotation during asymmetric cell division. *Nat Cell Biol* **3**, 50-57, doi:10.1038/35050558 (2001).
28 Minestrini, G., Harley, A. S. & Glover, D. M. Localization of Pavarotti-KLP in living Drosophila embryos suggests roles in reorganizing the cortical cytoskeleton during the mitotic cycle. *Mol Biol Cell* **14**, 4028-4038, doi:10.1091/mbc.E03-04-0214 (2003).
29 Johnson, K. G. *et al.* Axonal heparan sulfate proteoglycans regulate the distribution and efficiency of the repellent slit during midline axon guidance. *Current biology : CB* **14**, 499-504, doi:10.1016/j.cub.2004.02.005 (2004).

30 Wartlick, O. *et al.* Dynamics of Dpp signaling and proliferation control. *Science* **331**, 1154-1159, doi:10.1126/science.1200037 (2011).
31 Wucherpfennig, T., Wilsch-Brauninger, M. & Gonzalez-Gaitan, M. Role of Drosophila Rab5 during endosomal trafficking at the synapse and evoked neurotransmitter release. *J Cell Biol* **161**, 609-624, doi:10.1083/jcb.200211087 (2003).
32 Schindelin, J. *et al.* Fiji: an open-source platform for biological-image analysis. *Nature methods* **9**, 676-682, doi:10.1038/nmeth.2019 (2012).
